# Preoperative carbohydrate loading in elective colorectal surgery: postoperative complications and outcomes, a systematic review and meta-analysis

**DOI:** 10.1007/s00384-026-05125-7

**Published:** 2026-04-18

**Authors:** Aristotelis Nikitaras, Manousos-Georgios Pramateftakis, Konstantinos Perivoliotis, Sandra Maria Tsoti, Prokopis Christodoulou, Orestis Ioannidis, George Tzovaras

**Affiliations:** 1 1st Department of Surgery, Asklepieio General Hospital of Voula, Athens, Greece; 2 4th Department of Surgery, Aristotle University of Thessaloniki, Thessaloniki, Greece; 3 Department of Surgery, General Hospital of Volos, Volos, Greece; 4 Department of Surgery, Mediterraneo Hospital, Athens, Greece; 5 Department of Surgery, University Hospital of Larissa, Larissa, Greece

**Keywords:** Preoperative Care, Carbohydrates, Colorectal Surgery, Colectomy, Enhanced Recovery after Surgery

## Abstract

**Purpose:**

This study aimed to evaluate the clinical impact of preoperative carbohydrate loading in elective colorectal surgery.

**Methods:**

The review followed the Cochrane Handbook for Systematic Reviews of Interventions and PRISMA guidelines. A systematic search was conducted in MEDLINE (via PubMed), Scopus and Cochrane CENTRAL (via Wiley) to 1 August 2025. All randomised controlled trials (RCTs) investigating preoperative carbohydrate loading in elective colorectal surgery were assessed. The primary outcome was overall postoperative complications; secondary outcomes included specific types of complications, gastrointestinal recovery time, independent mobilisation, and length of stay (LOS).

**Results:**

A total of 3,483 citations were screened, yielding 13 RCTs with 996 patients (469 received preoperative carbohydrate loading, 198 consumed water and 329 followed overnight fasting). Carbohydrate loading did not significantly affect overall complications or mortality. Secondary outcomes suggested reduced respiratory infections (OR 0.35; *p* = 0.04), shorter time to first flatus (MD − 0.60; *p* < 0.01), earlier defecation (MD − 0.58; *p* = 0.01), shorter LOS (MD − 0.98; *p* < 0.01) and earlier mobilisation (MD − 0.49; *p* < 0.01).

**Conclusion:**

Preoperative carbohydrate loading was not associated with a statistically significant reduction in overall postoperative complications. Sensitivity analyses suggested limited robustness of the primary outcome. Secondary outcomes suggested faster gastrointestinal recovery, fewer respiratory infections, earlier mobilisation and shorter length of stay; however, these findings should be interpreted cautiously given heterogeneity across trials and variability in perioperative pathways. Further high-quality multicentre randomised controlled trials are needed to confirm these effects in contemporary ERAS pathways.

**Supplementary Information:**

The online version contains supplementary material available at 10.1007/s00384-026-05125-7.

## Introduction

Colorectal surgeries have been increasing in numbers in the last decades [1]. The rising demand of these operations highlights the importance of optimising preoperative care, with preoperative nutrition being one of its most critical components. Traditionally, patients were fasted for at least 12 h prior to surgery [2]. Nowadays, most surgeons have abandoned this approach and follow the Enhanced Recovery After Surgery (ERAS) protocols, which allow patients to drink clear fluids up to 2 h prior to surgery [3–5]. This is considered safe and does not increase perioperative aspiration risk. Certain ERAS protocols recommend the administration of carbohydrate drinks as clear fluids in the preoperative period, supported by evidence suggesting that this serves the patient’s best interest [3].

Carbohydrate drinks are clear fluids containing only carbohydrates as their sole energy source, usually at a concentration of 10–12.5%, and are characterized by low osmolality, which promotes rapid gastric emptying [6, 7]. They are commonly classified into two categories based on carbohydrate complexity: simple and complex. The simple carbohydrate category includes widely available beverages such as fruit juices and energy drinks, while the complex is mainly represented by specialised preoperative drinks [8]. There are multiple protocols for preoperative carbohydrate loading with different timing and number of doses, as well as volume of drink administered. However, most of the protocols are similar regarding the amount and timing of the last dose, which is 200–400 ml 2–3 h prior to surgery.

There is concrete evidence that preoperative fasting induces a catabolic state that is exacerbated by the upcoming surgical stress [9]. In contrast, preoperative carbohydrate loading promotes a postprandial anabolic state, characterized by reduced insulin resistance and enhanced insulin sensitivity [10]. This represents the optimal physiological state for patients undergoing major surgery, such as colorectal procedures, which are typically associated with increased insulin resistance and reduced insulin sensitivity [11–14]. Therefore, it is suggested that preoperative carbohydrate loading may represent a superior strategy in preoperative care, with the potential to enhance postoperative outcomes [3, 4, 7, 10, 11, 15–17].

Several studies have investigated preoperative carbohydrate loading in colorectal surgery; however, only one previous systematic review and meta-analysis has been published [18]. That review was limited to studies published up to 2019, permitted conference abstracts and prioritised glycaemic outcomes rather than clinical endpoints. In addition, the 2025 ERAS colorectal guidelines [5] downgraded the recommendation for preoperative carbohydrate loading from strong to weak, citing no clear clinical advantage over standard short fasting protocols. Here, we present a colorectal-specific systematic review and meta-analysis of full-text randomised controlled trials centred on overall postoperative complications, with predefined clinical secondary outcomes, to provide updated colorectal-specific estimates for contemporary practice and to support future guideline considerations.

## Methods

### Study protocol

This systematic review and meta-analysis was carried out according to the Cochrane Handbook for Systematic Reviews of Interventions and the Preferred Reporting Items for Systematic Reviews and Meta-Analyses (PRISMA) guidelines [19]. The review protocol has been registered on the Open Science Framework (OSF) Registries, 10.17605/OSF.IO/8V2YN.

### Outcomes

The primary outcome was overall postoperative complications. Secondary outcomes were specific postoperative complications, postoperative gastrointestinal recovery (time to first flatus and time to first defecation), time to independent mobilisation, and length of stay (LOS). Overall postoperative complications were analysed as the proportion of patients experiencing ≥ 1 complication within each trial’s follow-up window. Specific postoperative complications were extracted as defined by each trial, when reported. Gastrointestinal recovery and time to independent mobilisation were recorded in hours or days, as reported by each trial and were converted to days. LOS was recorded in days.

### Eligibility criteria

All randomised controlled trials (RCTs) investigating the role of preoperative carbohydrate loading in patients undergoing elective colorectal surgery were included. The exclusion criteria were: non-human studies, studies including paediatric population, non-English studies, studies with no outcome of interest and studies in the form of reviews, meta-analysis, conference abstracts, editorials, letters and expert opinions. A priori, we included colorectal-specific full-text randomised controlled trials and excluded trials framed as ‘abdominal surgery’ to minimise indirectness arising from heterogeneous surgical populations and perioperative pathways.

### Literature search

A thorough organised literature search was conducted on the following databases: MEDLINE (via PubMed), Scopus and Cochrane CENTRAL (via Wiley). The final literature search was conducted on Friday, 1 August 2025. The following search strategy was used: ("Preoperative Carbohydrate" OR "Oral Carbohydrate" OR Carbohydrate) AND ("Colorectal Surgery" OR Colectomy). Additionally, the manuscript reference list was also reviewed in case of relevant studies. Full reproducible search strategies for each database are provided in the Supplementary Material (Online Resource 1).

### Study selection and data collection

Study selection proceeded by removing duplicates, screening titles/abstracts, and assessing full texts against the eligibility criteria. Two reviewers (AN and SMT) independently and in duplicate screened titles/abstracts and full texts, extracted data, and assessed risk of bias. Disagreements were resolved by consensus, with unresolved cases adjudicated by a third reviewer (MGP). Risk of bias was evaluated using the Cochrane Risk of Bias 2 (RoB 2) tool [20].

### Statistical analysis

Statistical analyses were performed in IBM SPSS version 31 electronic software. Categorical outcomes were reported as odds ratios (OR) and continuous outcomes were reported as mean differences (MD), each with the respective 95% confidence intervals. If a trial did not provide the respective mean and standard deviation, then they were calculated from the reported median, range or interquartile range (IQR) [21]. Heterogeneity was estimated through the calculation of *I*^2^. The random effects (RE) or the fixed effects (FE) model was applied, based on the Cochran Q test results. More specifically, if Cochran Q test p value was below 0.1 then a RE model was introduced. The Restricted Maximum Likelihood (REML) estimator was applied in RE models. In terms of FE calculations, the Inverse Variance (IV) was introduced. Forest plots were generated for each outcome. To explore potential moderators of the primary outcome, subgroup and Fixed-Effects meta-regression analyses were conducted, with subgroup and bubble plots provided. A sensitivity (leave-one-out) analysis was also performed. Statistical significance was set at *p* < 0.05.

The funnel plot of the primary and secondary outcomes was inspected to identify the presence of publication bias. Egger’s test was applied for the primary outcome (overall complications).

## Results

### Study selection

**Fig. 1:**
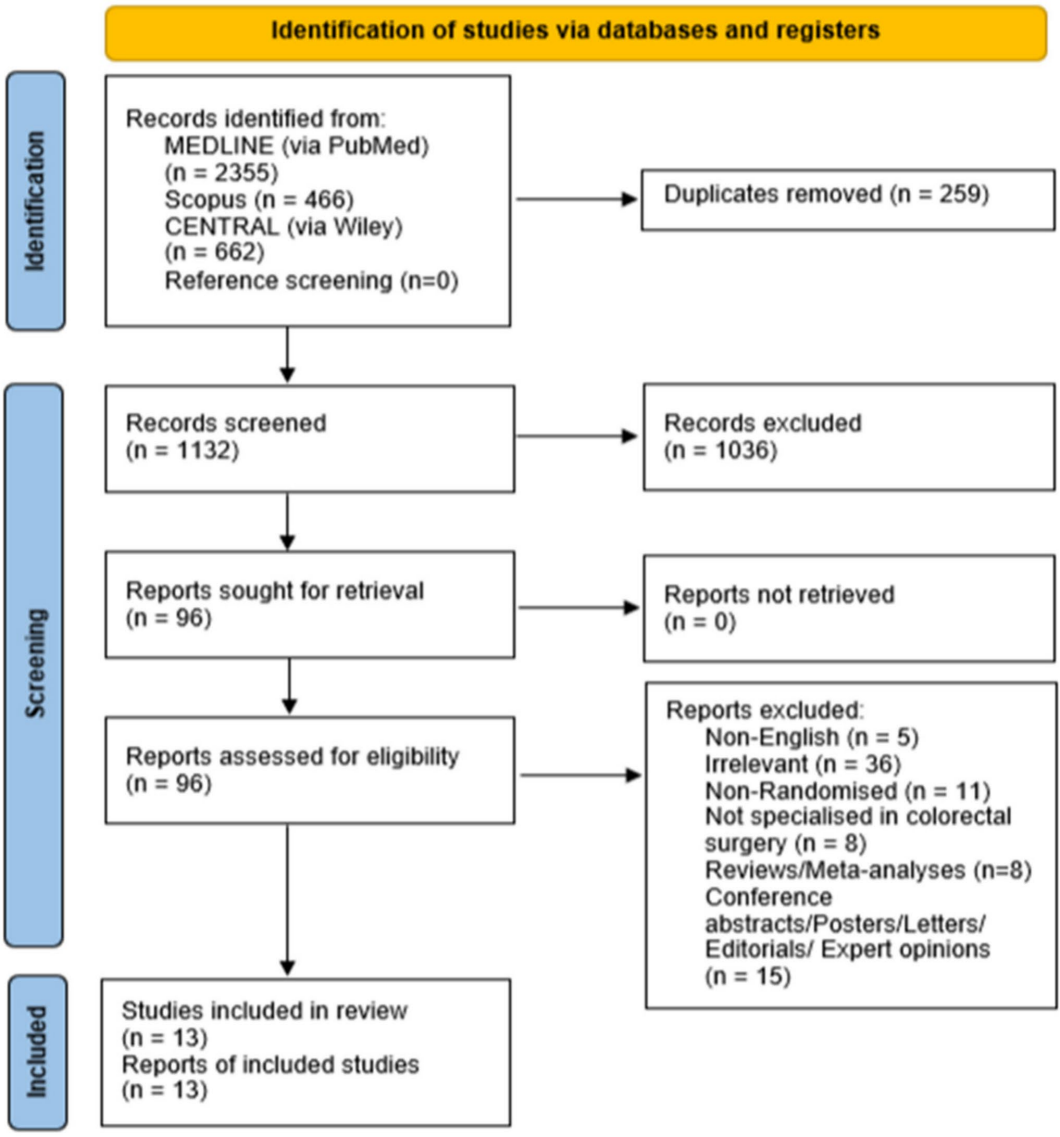
Preferred reporting items for systematic reviews flow diagram outlining the search algorithm results

The initial application of the search algorithm resulted in 3483 citations (MEDLINE via PubMed: 2355, Scopus: 466, Cochrane CENTRAL via Wiley: 662) (Fig. 1). After removing duplicates, 1132 articles remained. Title and abstract screening narrowed the selection to 96 articles, which were assessed in full text. A total of 83 articles were excluded due to eligibility criteria discordance (5 non-English studies, 11 non-RCTs, 36 irrelevant studies, 8 studies not focused on colorectal surgery, 8 reviews/meta-analyses, and 15 conference abstracts, letters, editorials, or expert opinions). Ultimately, 13 studies were included in the present systematic review and meta-analysis [8, 22–33].

### Study characteristics

The characteristics of the included RCTs are summarised in Table 1 and detailed further in Online Resource 2. The majority were prospective single-centre studies, with one multicentre (bicentric) study [8, 22–33]. Publication year ranged from 2006 [27] to 2024 [23]. Across all studies, a total of 996 patients were included. Patient demographics including sex, age, body mass index (BMI) and American Society of Anaesthesiologists (ASA) classification are detailed in Table 1. A discrepancy was noted in terms of the control group: four studies used water, eight studies implemented traditional overnight fasting and one study administered simple carbohydrate drinks. The follow-up period was stated in five studies, with four studies reporting a 30-day follow-up and one study reporting a 2-day follow-up. Only two studies [23, 25] used a classification system (Clavien-Dindo classification [34]) to categorise and state the postoperative complications.

**Table 1:** Characteristics of the included RCTs

Author	Country	Centre	Publication year	Study period	Groups	Control	Gender (M/F)	BMI	AGE	ASA SCORE
S. E. Noblett et al	United Kingdom	Single	2006	2005	1) CHO 2) Control	Fasting	N/A	N/A	1) 58 (30–77) 2) 59 (32–71)	I-IV
Z. G. Wang et al	China	Single	2010	2006–2007	1) CHO 2) Control	Fasting	1) 11/5 2) 9/7	1) 21 (19–24) 2) 23 (20–25)	1) 66 (48–74) 2) 63 (37–74)	I-III
M. Kaška et al	Czech Republic	Bicentric	2010	2008	1) Control 2) CHO	Fasting	122/99	20–30	60.4 (35–75)	I-II
P. Lidder et al	United Kingdom	Single	2013	2012	1) Control 2) CHO	Water	1) 15/15 2) 16/16	1) 25.7 (22.9,28.2) 2) 25.4 (21.9,29.0)	1) 73 (63.8–81) 2) 69 (64.0–73.8)	N/A
J. Webster et al	Australia	Single	2014	2011–2012	1) CHO 2) Control	Water	1) 8/14 2) 10/12	N/A	1) 54.5 (36–79) 2) 61.0 (27–77)	I-IV
H. Hamamoto et al	Japan	Single	2018	2013–2014	1) CHO 2) Control	Water	1) 17/14 2) 16/17	1) 21.3 (14.7–32.8) 2) 21.5 (16.7–26.2)	1) 71 (43–84) 2) 69 (49–85)	I-II
M. Wongyingsinn et al	Thailand	Single	2019	2014–2016	1) CHO 2) Control	Water	1) 17/17 2) 21/13	1) 22.1 ± 3.1 2) 23.2 ± 3.9	1) 62.9 ± 10.2 2) 62.7 ± 9.7	I-IV
N. Rizvanović et al	Bosnia and Herzegovina	Single	2019	2018	1) CHO 2) Control	Fasting	1) 13/12 2) 14/11	1) 26.4 ± 4.5 2) 24.7 ± 1.6	1) 60.2 ± 9.7 2) 61.0 ± 7.3	I-II
N. Karimian et al	Canada	Single	2020	2016–2017	1) Control 2) CHO	Simple CHO	1) 10/5 2) 7/7	1) 27.2 (5.5) 2) 25.5 (5.3)	1) 70 ± 10 2) 58 ± 13	I-IV
M. Shi et al	China	Single	2020	2017	1) CHO 2) Control	Fasting	1) 11/10 2) 11/10	1) 23 (20–25) 2) 22 (19–24)	1) 55 (36–74) 2) 56 (40–72)	I-IV
Y. Deng et al	China	Single	2022	2017–2018	1) CHO 2) Control	Fasting	1) 26/14 2) 21/21	1) 21.69 ± 3.33 2) 23.12 ± 3.35	1) 55.81 ± 13.83 2) 55.25 ± 13.45	I-III
N. Rizvanović et al	Bosnia and Herzegovina	Single	2023	2020–2022	1) CHO 2) Control	Fasting	1) 17/13 2) 16/14	1) 23.56 ± 1.51 2) 24.70 ± 1.68	1) 60.90 ± 6.80 2) 59.93 ± 9.31	I-III
S. M. Kumar et al	India	Single	2024	2021–2023	1) CHO 2) Control	Fasting	1) 17/19 2) 14/22	1) 23.10 ± 3.16 2) 23.92 ± 2.49	1) 52.20 ± 13.90 2) 56.60 ± 13.10	N/A

*M* Male, *F* Female, *BMI* Body Mass Index, *ASA* American Society of Anaesthesiologists, *CHO* Carbohydrate, *N/A* Not Available

There was considerable variability in the type and concentration of carbohydrate drinks administered. Specifically, ten trials used complex carbohydrate drinks (intervention) [22–25, 27–29, 31–33], one used simple carbohydrate drinks in one arm (control) and complex in the other (intervention) [8], and in two trials the type of carbohydrate drink was not specified [26, 30]. Volumes and timing of administration also varied across studies.

The colorectal procedures included across the studies were heterogeneous. Six studies included only colorectal cancer operations [22–25, 28, 31], one study had exclusively colon cancer operations [30], three studies incorporated operations for benign and malignant diseases of the colon [8, 32, 33], and three studies did not specify their procedures [26, 27, 29]. Moreover, six studies included only open surgeries, three studies focused on minimally invasive surgeries (MIS) and four studies had both types. In total, 672 (67.5%) open and 324 (32.5%) minimally invasive surgeries were included.

### Risk of bias within studies

**Fig. 2:**
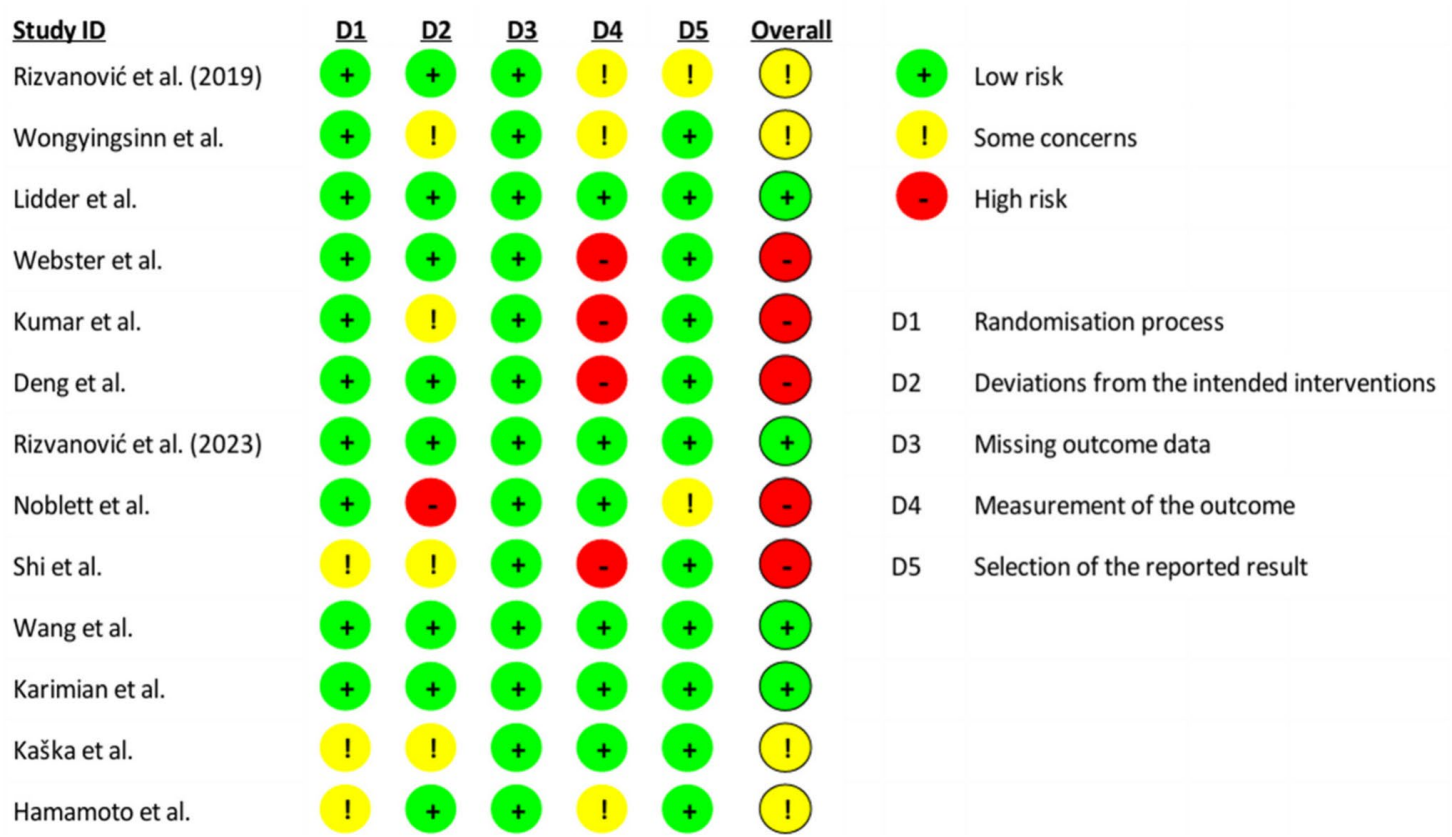
Risk of bias within studies

Quality evaluation of the included studies was performed using the risk of bias tool RoB 2. Four studies were graded as low risk; four studies were graded as some concerns (medium risk) and five studies were graded as high risk (Fig. 2). Additionally, Cohen’s Kappa for inter-rater agreement was *k* = 0.90 (*p* < 0.001).

### Risk of bias across studies

Funnel plots were inspected for the primary and secondary outcomes to assess the presence of publication bias. Additionally, Egger’s test for overall complications was performed (0.6 [95% CI: –2.3, 3.6], *p* = 0.656).

### Overall postoperative complications

**Fig. 3:**
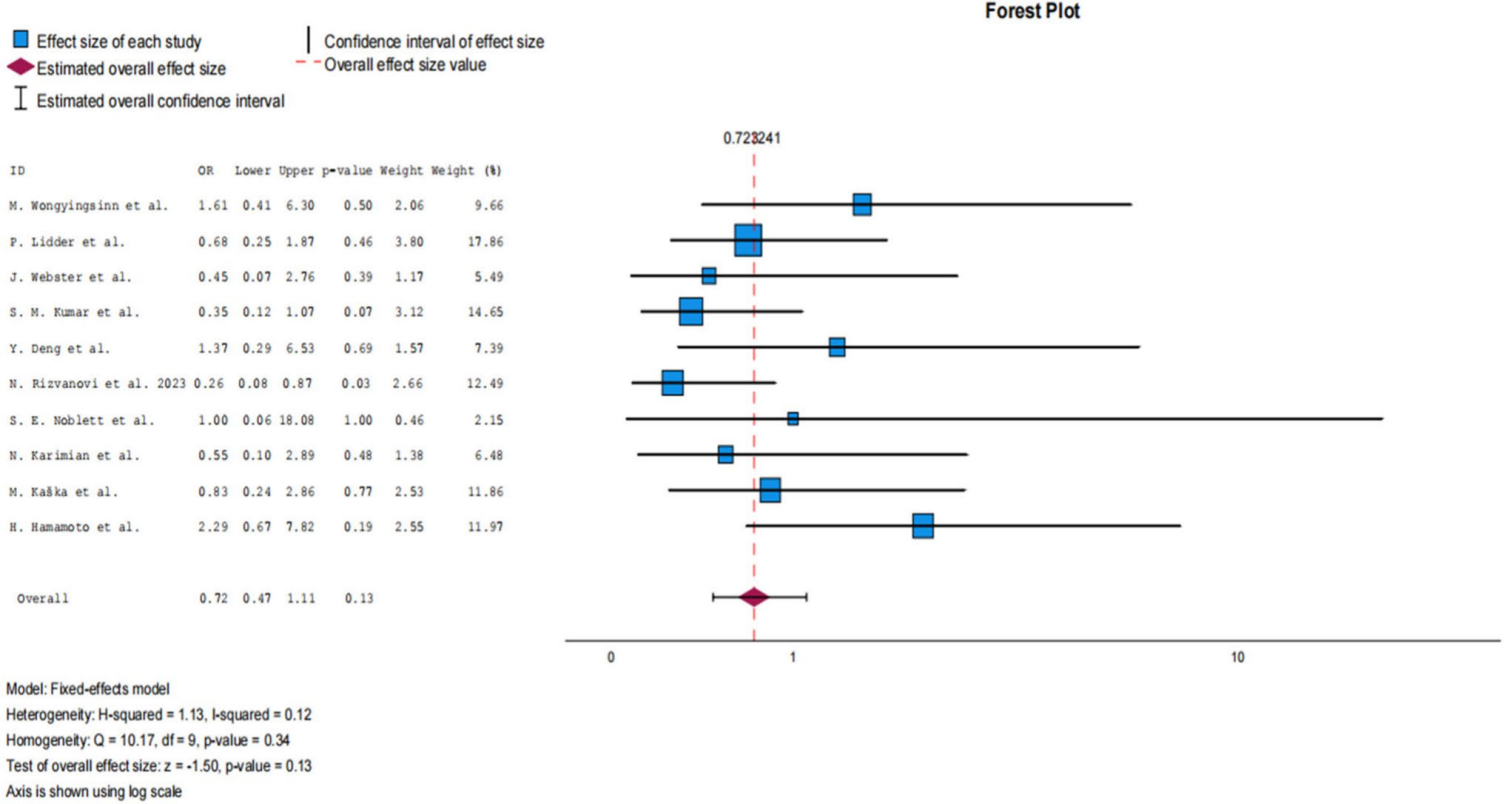
Forest plot for primary outcome (overall postoperative complications)

Regarding the primary outcome, ten studies encompassing a total of 839 patients investigated the postoperative complications. The meta-analysis (Table 2) demonstrated no statistically significant difference between the intervention and control groups in overall postoperative complications (OR 0.72; 95% CI: 0.47 to 1.11; *p* = 0.13) with low heterogeneity (*I*^2^ = 12%, *p* = 0.34). To explore heterogeneity in the primary outcome, subgroup analyses by control type, follow-up period (30-day vs non–30-day follow-up) and by number of centres (single vs multicentre) did not demonstrate significant differences in the effect of preoperative carbohydrate loading on overall complications (Online Resource 6). Similarly, meta-regression analyses showed no significant association between study-level characteristics (publication year, percentage of male patients, mean BMI, mean age, or percentage of minimally invasive procedures) and the primary outcome (Online Resource 5). Leave-one-out sensitivity analysis showed that exclusion of the Hamamoto et al. study resulted in a statistically significant pooled effect for overall postoperative complications, indicating sensitivity of the primary outcome to this individual study (Online Resource 8). Additionally, no significant difference was noted regarding mortality (OR 0.32, 95% CI: 0.03 to 3.18; *p* = 0.33). Forest plot for overall postoperative complications can be seen at Fig. 3.

**Table 2:** Statistical analysis of primary and secondary outcomes

Outcome	Statistical method	Effect estimate 95% CI	p	I^2^	Heterogeneity p
Overall complications	Fixed Effects	0.72[0.47, 1.11]	0.13	12%	0.34
Anastomotic leakage	Fixed Effects	0.49[0.17, 1.45]	0.2	0%	0.96
Surgical site infections	Fixed Effects	0.98[0.47, 2.05]	0.95	0%	0.8
Respiratory infections	Fixed Effects	0.35[0.13, 0.94]	0.04	0%	0.63
Ileus	Fixed Effects	1.96[0.67, 5.77]	0.22	0%	0.82
Thromboembolic complications	Fixed Effects	0.33[0.06, 1.71]	0.19	0%	0.87
Cardiac complications	Fixed Effects	1.83[0.55, 6.06]	0.32	0%	0.69
Septic complications	Fixed Effects	0.59[0.07, 4.96]	0.63	0%	0.63
Mortality	Fixed Effects	0.32[0.03, 3.18]	0.33	0%	1
Time to first flatus	Fixed Effects	−0.60[−0.75, −0.45]	< 0.01	33%	0.21
Time to first defecation	Random Effects	−0.58[−1.0, −0.16]	0.01	76%	< 0.001
Time to mobilisation	Fixed Effects	−0.49[−0.69, −0.29]	< 0.01	14%	0.31
Length of stay	Random Effects	−0.98[−1.59, −0.38]	< 0.01	75%	< 0.001

### Secondary outcomes

Analyses of the remaining secondary outcomes demonstrated significant benefits in favour of the carbohydrate-loading groups. Specifically, patients receiving preoperative carbohydrate drinks experienced shorter time to first flatus (MD −0.60; 95% CI: −0.75 to −0.45; *p* < 0.01), earlier first defecation (MD −0.58; 95% CI: −1.00 to −0.16; *p* = 0.01), reduced length of hospital stay (MD −0.98; 95% CI: −1.59 to −0.38; *p* < 0.01) and a significant reduction in time to independent mobilisation (MD −0.49; 95% CI: −0.69 to −0.29; *p* < 0.01).

**Fig.4:**
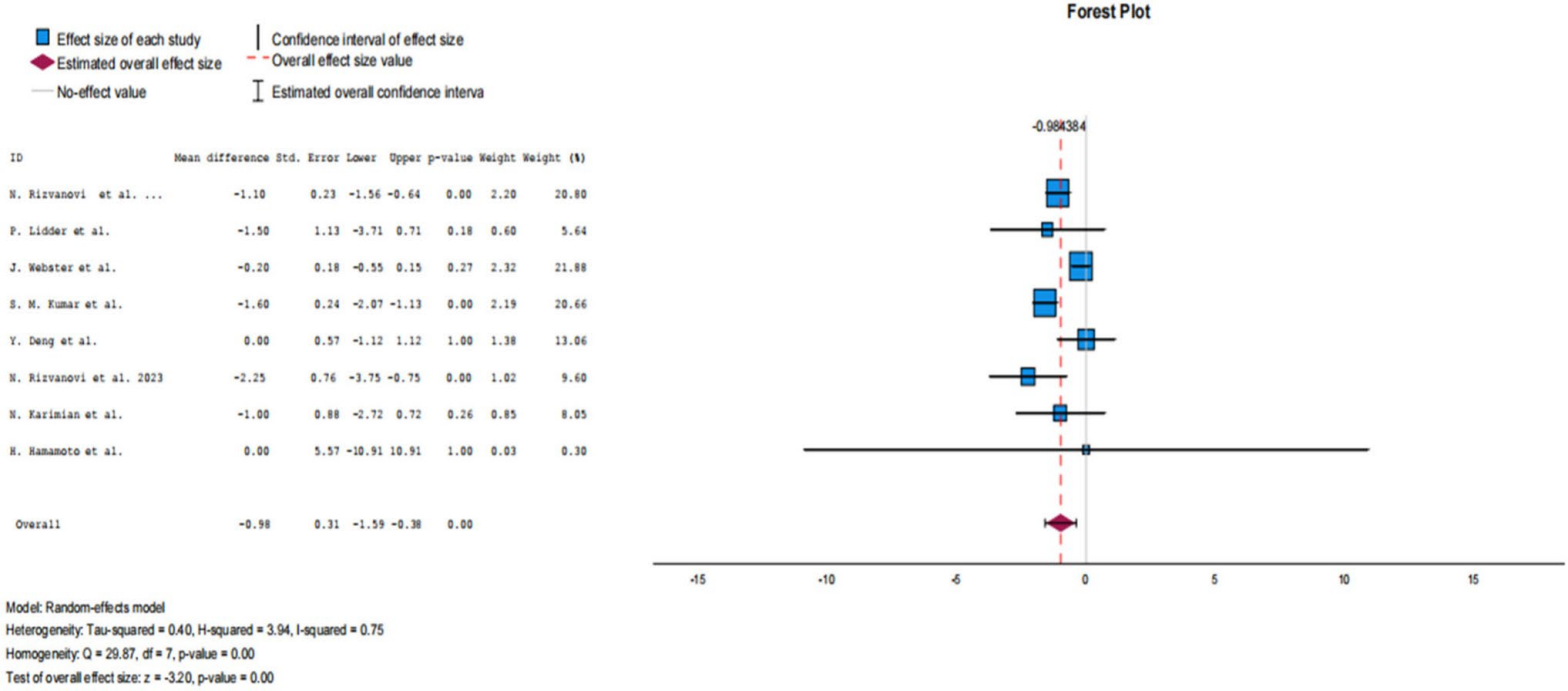
Forest plot for length of stay

Forest plot for length of stay is also presented in the main manuscript (Fig. 4); remaining forest plots are provided in Online Resource 3, with corresponding funnel plots in Online Resource 4.

## Discussion

In modern surgical practice, traditional preoperative overnight fasting has been replaced by ERAS protocols, which recommend allowing patients to consume water up to two hours before surgery, and in some cases, clear fluids including carbohydrate drinks [3, 5]. Preoperative carbohydrate loading has been investigated extensively due to its potential to induce a favourable postprandial anabolic state that counters the catabolic stress response triggered by surgery. Several studies have supported its benefits in improving postoperative inflammation markers and insulin sensitivity, thereby underpinning a growing clinical interest in its use [8, 22–24, 28, 29, 31, 35–37].

This systematic review and meta-analysis synthesised the existing RCTs investigating the impact of preoperative carbohydrate loading on postoperative outcomes in elective colorectal surgery. Our findings indicate no statistically significant differences in overall postoperative complications or mortality between patients who received carbohydrate loading and those who did not. Notably, when analysing complications individually, we found a statistically significant reduction in respiratory infections in patients receiving carbohydrate loading. A potential biological explanation is attenuation of perioperative insulin resistance and inflammatory stress, which has been associated with improved immune function in experimental and clinical studies [37, 38]. However, given the limited number of contributing trials and the exploratory nature of secondary comparisons, this finding should be interpreted cautiously. Furthermore, our analysis demonstrated statistically significant improvements in gastrointestinal recovery parameters, including shorter times to first flatus and defecation, reduced LOS and a significant decrease in time to independent mobilisation. However, these outcomes were associated with variable heterogeneity (*I*^2^ = 14–76%), likely reflecting differences in study protocols, ERAS adherence, type of carbohydrate drink used, and dosage and timing of administration. These sources of heterogeneity underscore the need for cautious interpretation and highlight a critical area for future research standardisation.

One included RCT [8] compared simple versus complex carbohydrate drinks rather than carbohydrate loading versus fasting or water. To ensure this different clinical contrast did not influence the main findings, we assessed its impact in sensitivity analyses; exclusion of this trial did not materially change the pooled estimate for overall postoperative complications.

The only prior meta-analysis on preoperative carbohydrate loading in colorectal surgery was published by Lu et al. (2022) [18]. That study included 12 RCTs (758 patients), permitted conference abstracts and prioritised glycaemic outcomes; it reported shorter time to flatus/defecation and reduced length of stay, without differences in morbidity or patient-reported well-being. Our review adds to the literature by providing an updated, colorectal-specific synthesis restricted to full-text randomised controlled trials, with overall postoperative complications prespecified as the primary outcome and predefined patient-centred secondary outcomes. We included 13 full-text colorectal-specific RCTs with nearly 1,000 patients. Specifically, compared with Lu et al. [18], we excluded four studies they included (two conference abstracts and two trials framed as ‘abdominal surgery’) to reduce indirectness and incomplete reporting, and we added five additional colorectal-specific randomised controlled trials. While overall complications and mortality did not differ significantly, leave-one-out sensitivity analysis demonstrated that exclusion of the Hamamoto et al. study altered the statistical significance of the pooled estimate, indicating limited robustness of the primary outcome. Secondary outcomes showed statistically significant improvements in gastrointestinal recovery, length of stay, respiratory infections and mobilisation; however, these findings are exploratory and hypothesis-generating, given the non-significant primary outcome, variable heterogeneity, and the absence of adjustment for multiple comparisons. This indicates that the overall pooled estimate for the primary outcome should be interpreted cautiously, as the statistical significance is not stable and can be influenced by individual studies. By restricting inclusion to full-text colorectal-specific RCTs and expanding the patient pool, our analysis provides updated colorectal-focused effect estimates that contribute additional context to the existing literature.

Interestingly, the most recent ERAS colorectal guidelines (2025) downgraded the recommendation for preoperative carbohydrate loading from strong to weak, citing improved insulin sensitivity but no clear clinical advantage over standard short fasting protocols [5]. In our colorectal-specific analysis of full-text randomised controlled trials, carbohydrate loading was associated with improvements in several patient-centred endpoints, including faster gastrointestinal recovery, earlier mobilisation, fewer respiratory infections and shorter length of stay, despite no statistically significant reduction in overall postoperative complications. These findings provide additional colorectal-specific estimates that may inform ongoing discussion regarding the role of carbohydrate loading within ERAS pathways. Further large multicentre RCTs within modern ERAS pathways are warranted to confirm these benefits and to define the patient populations most likely to gain from this intervention.

The interpretation of this systematic review and meta-analysis is subject to several limitations. Generalisability is restricted by the exclusion criteria applied in most included RCTs, which largely omitted patients with diabetes mellitus, advanced age, extreme body mass indices, pregnancy, delayed gastric emptying, disseminated disease or metabolic disorders. Therefore, caution is required when extrapolating the findings to broader surgical populations. Many trials were small in size, limiting statistical power and contributing to heterogeneity, and only two studies used a structured complication classification (Clavien–Dindo classification), while others used heterogeneous definitions of postoperative complications, further reducing comparability across studies. As multiple secondary outcomes were analysed without formal adjustment for multiplicity, isolated statistically significant findings may be vulnerable to type I error. At the review level, the analysis was limited to English-language publications, and no protocol was prospectively registered. Taken together, these factors indicate that the overall certainty of evidence for secondary outcomes should be considered limited and hypothesis-generating rather than definitive.

## Conclusion

This systematic review and meta-analysis did not demonstrate a statistically significant reduction in overall postoperative complications with preoperative carbohydrate loading in elective colorectal surgery. Sensitivity analyses indicated that the primary outcome was influenced by individual studies, highlighting limited robustness and uncertainty regarding the necessity of preoperative carbohydrate loading to prevent complications. Secondary outcomes suggested potential improvements in gastrointestinal recovery, length of stay, respiratory infections, and mobilisation; however, these findings are context-dependent and should be interpreted cautiously given heterogeneity across trials, variability in perioperative pathways, and limited robustness of some pooled estimates. Further large, well-designed multicentre randomised controlled trials within modern ERAS programmes are warranted to confirm whether these secondary effects translate into consistent clinical benefit and to clarify the impact on overall postoperative complications with greater robustness.

## Supplementary Information

Below is the link to the electronic supplementary material.

Supplementary file1 Search strategies (PDF 61.0 KB)

Supplementary file2 Supplementary tables (study characteristics, postoperative complications, gastrointestinal recovery and LOS, statistical analyses of outcomes, explanatory analyses) (PDF 222 KB)

Supplementary file3 Forest plots for all outcomes (PDF 771 KB)

Supplementary file4 Funnel plots for all outcomes (PDF 1.12 MB)

Supplementary file5 Meta-regression bubble plots (age, BMI, male percentage, MIS percentage, publication year) (PDF 578 KB)

Supplementary file6 Subgroup plots (control type, number of centres) (PDF 220 KB)

Supplementary file7 PRISMA 2020 checklist (PDF 231 KB)

Supplementary file8 Sensitivity analyses (leave-one-out) and follow-up duration subgroup forest plot (30-day follow-up vs non–30-day follow-up) (PDF 157 KB)

## Data Availability

Extracted dataset and any additional materials are available from the corresponding author on reasonable request. The review protocol is registered on the Open Science Framework (10.17605/OSF.IO/8V2YN).
