# Supplementary material for: Preoperative carbohydrate loading in elective colorectal surgery: postoperative complications and outcomes, a systematic review and meta-analysis
Source: Int J Colorectal Dis. 2026 Apr 18;41(1):95. doi: 10.1007/s00384-026-05125-7 (PMC13222900; doi:10.1007/s00384-026-05125-7)
Supplement: Supplementary file 1 — Supplementary file1 Search strategies (PDF 61.0 KB) [file 384_2026_5125_MOESM1_ESM.pdf]

Article title: Preoperative carbohydrate loading in elective colorectal surgery: postoperative complications and outcomes, a systematic review and meta-analysis

Journal: International Journal of Colorectal Disease

Authors: Aristotelis Nikitaras, Manousos-Georgios Pramateftakis, Konstantinos Perivoliotis, Sandra Maria Tsoti, Prokopis Christodoulou, Orestis Ioannidis, George Tzovaras

Corresponding author: Aristotelis Nikitaras, 1st Department of Surgery, Asklepieio General Hospital of Voula, Athens, Greece

Email: nikitaras.aristotelis@gmail.com

Online Resource 1: Search strategies

MEDLINE (via PubMed): The following search algorithm was entered into the PubMed search interface using standard search settings: (“Preoperative Carbohydrate” OR “Oral Carbohydrate” OR Carbohydrate) AND (“Colorectal Surgery” OR Colectomy).

Scopus: The same search algorithm was entered into the Scopus search interface using standard search settings.

Cochrane CENTRAL (via Wiley): The same search algorithm was entered into the Cochrane CENTRAL search interface using standard search settings.

The final literature search was conducted on 1 August 2025. No date restrictions were applied. Language restrictions were applied at the study selection stage as described in the eligibility criteria.
