# Supplementary material for: Preoperative carbohydrate loading in elective colorectal surgery: postoperative complications and outcomes, a systematic review and meta-analysis
Source: Int J Colorectal Dis. 2026 Apr 18;41(1):95. doi: 10.1007/s00384-026-05125-7 (PMC13222900; doi:10.1007/s00384-026-05125-7)
Supplement: Supplementary file 2 — Supplementary file2 Supplementary tables (study characteristics, postoperative complications, gastrointestinal recovery and LOS, statistical analyses of outcomes, explanatory analyses) (PDF 222 KB) [file 384_2026_5125_MOESM2_ESM.pdf]

Article title: Preoperative carbohydrate loading in elective colorectal surgery: postoperative complications and outcomes, a systematic review and meta-analysis

Journal: International Journal of Colorectal Disease

Authors: Aristotelis Nikitaras, Manousos-Georgios Pramateftakis, Konstantinos Perivoliotis, Sandra Maria Tsoti, Prokopis Christodoulou, Orestis Ioannidis, George Tzovaras

Corresponding author: Aristotelis Nikitaras, 1st Department of Surgery, Asklepieio General Hospital of Voula, Athens, Greece

Email: nikitaras.aristotelis@gmail.com

Online Resource 2: Supplementary Tables (S2-S6)

Table S2: Surgical operations

**Author**	**Sample**	**Cases**	**Groups**	**Control**	**TNM Stage**	**Open / MIS**	**Colon/Rectum/Not Specified**	**Right Colectomy**	**Left Colectomy**	**Sigmoidectomy**	**LAR**	**APR**	**Proctocolectomy**	**Other - Not Specified**
N. Rizvanović et al.	50	Colorectal Cancer (100%)	1) CHO<br>2) Control	Fasting	N/A	Open	**Total: 24/26/0**<br>1) 13/12<br>2) 11/14	1) 9<br>2) 8	-	HARTMANN'S<br>1) 4<br>2) 3	1) 7<br>2) 9	1) 4<br>2) 5	1) 1<br>2) 0	-
M. Wongyingsinn et al.	68	Colorectal Cancer (91%)	1) CHO<br>2) Control	Water	N/A	**Total:38/30**<br>1) 21/13<br>2) 17/17	**Total: 31/37/0**<br>1) 18/16<br>2) 13/21	N/A	N/A	N/A	N/A	N/A	N/A	N/A
P. Lidder et al.	120	Colorectal Cancer (100%)	1)Control<br>2) CHO	Water	N/A	**Total:93/27**<br>1) 19/11<br>2) 27/5	**Total: 45/75/0**<br>1) 9/21<br>2) 15/17	1) 9<br>2) 13	1) 0<br>2) 0	1) 0<br>2) 2	1) 21<br>2) 17	-	-	-
J. Webster et al.	44	Colorectal Cancer (59%)	1) CHO<br>2) Control	Clear fluids	N/A	**Total:7/37**<br>1)3/19<br>2)4/18	**Total: 6/14/24**<br>1) 4/13/11<br>2) 2/11/17	**Colectomy**<br>1) 3<br>2) 2		1) 1<br>2) 0	1) 11<br>2) 7	-	1) 2<br>2) 4	1) 11<br>2) 17
S. M. Kumar et al.	72	Colorectal Cancer (100%)	1) CHO<br>2) Control	Fasting	N/A	**Total:57/15**<br>1)27/9<br>2)30/6	**Total: 39/32/1**<br>1) 20/15/1<br>2) 19/17	-	1) 2<br>2) 5	1) 7<br>2) 4	1) 2<br>2) 5	1) 13<br>2) 12	-	1) 1<br>2) 0
Y. Deng et al.	122	Colorectal (100%) Types of cases - N/A	1) CHO<br>2) Control	Fasting	N/A	MIS	**Total: 88/34/0**<br>1) 30/12<br>2) 29/11<br>3) 29/11	N/A	N/A	N/A	N/A	N/A	N/A	N/A
N. Rizvanović et al.	60	Colorectal Cancer (100%)	1) CHO<br>2) Control	Fasting	N/A	Open	**Total: 19/41/0**<br>1) 10/20<br>2) 9/21	N/A	N/A	N/A	N/A	N/A	N/A	N/A
S. E. Noblett et al.	35	Colorectal (100%) Types of cases - N/A	1) CHO<br>2) Control	Fasting	N/A	Open	**Total: 12/18/5**<br>1) 5/5/2<br>2) 4/8/1	1) 4<br>2) 3	-	1) 1<br>2) 1	1) 5<br>2) 6	1) 0<br>2) 1	1) 0<br>2) 1	1) 2<br>2) 1
M. Shi et al.	63	Colorectal Cancer (100%)	1) CHO<br>2) Control	Fasting	1) I+II(4), III(17)<br>2)I+II(3), III(18)	Open	**Total: 35/28**<br>1) 12/9<br>2) 11/10	1) 7<br>2) 8	1) 2<br>2) 1	1) 3<br>2) 2	1) 9<br>2) 10	-	-	-
Z. G. Wang et al.	48	Colorectal Cancer (100%)	1) CHO<br>2) Control	Fasting	1)I(2),II(11),III(3)<br>2)I(2),II(10),III(4)	Open	**Total: 27/21**<br>1) 8/8<br>2) 10/6	**Colectomy**<br>1) 4<br>2) 4		1) 4<br>2) 6	1) 3<br>2) 4	1) 5<br>2) 2	-	-
N. Karimian et al.	29	Colorectal Cancer (100%)	1)Control<br>2) CHO	Simple CHO	N/A	MIS	**Only Colon (29)**<br>1) 15<br>2) 14	1) 5<br>2) 7	1) 2<br>2) 0	1) 8<br>2) 6	-	-	-	1)0<br>2)1
M. Kaška et al.	221	Colorectal (100%) Types of cases - N/A	1)Control<br>2) CHO	Fasting	N/A	Open	**Not Specified (221)**	N/A	N/A	N/A	N/A	N/A	N/A	N/A
H. Hamamoto et al.	64	Colon Cancer (100%)	1) CHO<br>2) Control	Water	1)0(3),I(12),II(4),III(10),IV(2)<br>2)0(6), I(8), II(7),III(11),IV(1)	MIS	**Total: 56/8**<br>1)28/3<br>2)28/5	1)12<br>2)16	1)5<br>2)4	1)11<br>2)8	1)3<br>2)5	-	-	-

CHO: Carbohydrate, N/A: Not Available, TNM: Tumor-Nodes-Metastasis, MIS: Minimally Invasive Surgery, LAR: Low Anterior Resection, APR: Abdominoperineal Resection

Table S3: Postoperative complications

Author	Groups	Control	Sample	Follow-up	Complications	Clavien-Dindo Classification (Grades)				
						I	II	III	IV	V
N. Rizvanović et al.	1) CHO<br>2)Control	Fasting	1) 25<br>2) 25	POD 2	N/A	N/A	N/A	N/A	N/A	N/A
M. Wongyingsinn et al.	1) CHO<br>2)Control	Water	1) 34<br>2) 34	POD 30	1) 6 (17,6%)<br>2) 4 (11.7%)	N/A	N/A	N/A	N/A	N/A
P. Lidder et al.	1)Control<br>2)CHO	Water	1) 30<br>2) 32	POD 30	1) 15 (50%)<br>2) 13 (41%)	N/A	N/A	N/A	N/A	N/A
J. Webster et al.	1) CHO<br>2)Control	Water	1) 22<br>2) 22	N/A	1) 2 (9,1%)<br>2) 4 (18,1%)	N/A	N/A	N/A	N/A	N/A
S. M. Kumar et al.	1) CHO<br>2)Control	Fasting	1) 36<br>2) 36	N/A	1) 6 (17%)<br>2) 13 (36%)	1) 16,7%<br>2) 36,1%		1) -<br>2) -		
Y. Deng et al.	1) CHO<br>2)Control	Fasting	1) 40<br>2) 42	N/A	1) 4 (9,6%)<br>2) 3 (7,5%)	N/A	N/A	N/A	N/A	N/A
N. Rizvanović et al.	1) CHO<br>2)Control	Fasting	1) 30<br>2) 30	POD 30	1) 5 (16,7%)<br>2)13(43,3%)	1) 10%<br>2) 36,7%	1) 6,7%<br>2) 50%	1) -<br>2) 16,7%	1) -<br>2) 16,7%	1) -<br>2) 3,3%
S. E. Noblett et al.	1) CHO<br>2)Control	Fasting	1) 12<br>2) 12	N/A	1) 1 (8,3%)<br>2) 1 (8.3%)	N/A	N/A	N/A	N/A	N/A
M. Shi et al.	1) CHO<br>2)Control	Fasting	1) 21<br>2) 21	N/A	N/A	N/A	N/A	N/A	N/A	N/A
Z. G. Wang et al.	1) CHO<br>2)Control	Fasting	1) 16<br>2) 16	N/A	N/A	N/A	N/A	N/A	N/A	N/A
N. Karimian et al.	1)Control<br>2)CHO	Simple CHO	1) 15<br>2) 14	POD 30	1) 5 (33,3%)<br>2) 3 (21,4%)	N/A	N/A	N/A	N/A	N/A
M. Kaška et al.	1)Control<br>2)CHO	Fasting	1) 75<br>2) 74	N/A	1) 6 (8%)<br>2) 5 (6,6%)	N/A	N/A	N/A	N/A	N/A
H. Hamamoto et al.	1) CHO<br>2)Control	Water	1) 31<br>2) 33	Short-term	1) 9 (29%)<br>2) 5 (15,2%)	N/A	N/A	N/A	N/A	N/A

CHO: Carbohydrate, Active: Polymeric Supplement, POD: Post-Operative Day, N/A: Not Available

Table S4: Remaining outcomes

AUTHOR	Groups	Control	TIME TO 1ST FLATUS	TIME TO DEFECATION	TIME TO INDEPENDENT AMBULATION	LENGTH OF STAY
Rizvanović, N *et al.*	1) CHO<br>2) Control	Fasting	**DAYS**<br>1) 2.5±0.5 (CHO)<br>2) 3.1±0.5 (FAST)	**DAYS**<br>1) 3.2±0.4<br>2) 4.0±0.9	**DAYS**<br>1) 3.6±0.4<br>2) 4.2±0.5	1) 7.7 ±0.4<br>2) 8.8 ±1.1
Mingkwan Wongyingsinn et al.	1) CHO<br>2) Control	Water	NOT STUDIED	NOT STUDIED	2MIN AND 6MIN WALKING-TESTS	Median Duration<br>1) 5,5 (CHO)<br>2) 6 (CONTROL)
P. Lidder et al.	1)Control<br>2) CHO	Water	NO DIFFERENCE IN RETURN OF BOWEL FUNCTION		NOT MENTIONED	1) 8.5 (6.0, 13.3)<br>2) 7.0 (6.0, 10.5)
JOAN WEBSTER et al.	1) CHO<br>2) Control	Clear Fluids	**HOURS**<br>1) 34.7 (26.0 -46.1)<br>2) 49.8 (33.4 - 74.2)	**HOURS**<br>1) 46.5 (33.2 - 65.2)<br>2) 68.4 (50.6 - 92.5)	NOT STUDIED	1) 4.1 (3.2 - 5.4)<br>2) 4.3 (3.2 - 5.7)
Shanmugam Mannoj Kumar et al.	1) CHO<br>2) Control	Fasting	RETURN OF GASTROINTESTINAL FUNCTION<br>1) 4.08 ± 0.80<br>2) 4.19 ± 0.90		1) 5.3 ± 1.8<br>2) 5.8 ± 1.4	1) 7.0 ± 0.8<br>2) 8.6 ± 1.2
Yingqing Deng, MD et al.	1) CHO<br>2) Control	Fasting	**HOURS**<br>1) 40.50 (27.00-64.25)<br>2) 43.00 (30.13-63.50)	**HOURS**<br>1) 78.00(41.25-116.38)<br>2) 72.50(59.00-105.00)	**HOURS**<br>1) 27.00(23.00-44.75)<br>2)33.00(25.00-44.38)	1) 8.00 (6.00-10.00)<br>2) 8.00 (6.00-9.00)
Nermina Rizvanovića et al.	1) CHO<br>2) Control	Fasting	NOT STUDIED	NOT STUDIED	NOT STUDIED	1) 7.61 ±2.34<br>2) 9.86 ±3.47
S. E. Noblett et al.	1) CHO<br>2) Control	Fasting	**DAYS**<br>1) 2<br>2) 3	**DAYS**<br>1) 2<br>2) 3,5	NOT MENTIONED	1) 7,5<br>2) 10
Mengyao Shi	1) CHO<br>2) Control	Fasting	NOT STUDIED	NOT STUDIED	NOT STUDIED	NOT STUDIED
Z. G. Wang et al.	1) CHO<br>2) Control	Fasting	NOT STUDIED	NOT STUDIED	NOT STUDIED	NOT STUDIED
Negar Karimian, MD et al.	1)Control<br>2) CHO	Simple CHO	NOT STUDIED	NOT STUDIED	NOT STUDIED	1) 3.5 (3–7)<br>2) 2.5 (2–4)
Milan Kaška et al.	1)Control<br>2) CHO	Fasting	NOT STUDIED	NOT STUDIED	NOT STUDIED	1) 11<br>2) 11
Hiroki Hamamoto et al.	1) CHO<br>2) Control	Water	**DAYS**<br>1) 1 (1–7)<br>2) 2 (1–6)	**DAYS**<br>1) 3 (1–8)<br>2) 4 (1–8)	NOT STUDIED	1) 10 (7–122)<br>2) 10 (6–61)
CHO: Carbohydrate						

Table S5: Statistical analysis of primary and secondary outcomes

Outcome	Statistical Method	Effect Estimate 95%CI	p	I^2	Heterogeneity p
Overall Complications	Fixed Effects	0.72[0.47, 1.11]	0.13	12%	0.34
Anastomotic Leakage	Fixed Effects	0.49[0.17, 1.45]	0.2	0%	0.96
Surgical Site Infections	Fixed Effects	0.98[0.47, 2.05]	0.95	0%	0.8
Respiratory Infections	Fixed Effects	0.35[0.13, 0.94]	0.04	0%	0.63
Ileus	Fixed Effects	1.96[0.67, 5.77]	0.22	0%	0.82
Thromboembolic Complications	Fixed Effects	0.33[0.06, 1.71]	0.19	0%	0.87
Cardiac Complications	Fixed Effects	1.83[0.55, 6.06]	0.32	0%	0.69
Septic Complications	Fixed Effects	0.59[0.07, 4.96]	0.63	0%	0.63
Mortality	Fixed Effects	0.32[0.03, 3.18]	0.33	0%	1
Time to First Flatus	Random Effects	-0.94[-1.64, -0.25]	0.01	83%	<0.001
Time to First Defecation	Random Effects	-0.77[-1.49, -0.05]	0.04	88%	<0.001
Time to Mobilisation	Random Effects	-0.6[-1.2, -0.01]	0.05	77%	0.01
LOS	Random Effects	-0.58[-1, -0.17]	0.01	79%	<0.001
LOS: Length of Stay					

Table S6: Explanatory Analyses of Primary Outcome

Variable	Type of Sub-Analysis	p
Control Type	Subgroup	0.29
Number of Centers	Subgroup	0.81
30 days Follow Up	Subgroup	0.47
Publication Year	Meta-Regression	0.348
Males Percentage	Meta-Regression	0.296
BMI	Meta-Regression	0.134
Age	Meta-Regression	0.209
MIS Percentage	Meta-Regression	0.103
BMI: Body Mass Index, MIS: Minimally Invasive Surgery		
