# Supplementary material for: Preoperative carbohydrate loading in elective colorectal surgery: postoperative complications and outcomes, a systematic review and meta-analysis
Source: Int J Colorectal Dis. 2026 Apr 18;41(1):95. doi: 10.1007/s00384-026-05125-7 (PMC13222900; doi:10.1007/s00384-026-05125-7)
Supplement: Supplementary file 3 — Supplementary file3 Forest plots for all outcomes (PDF 771 KB) [file 384_2026_5125_MOESM3_ESM.pdf]

Article title: Preoperative carbohydrate loading in elective colorectal surgery: postoperative complications and outcomes, a systematic review and meta-analysis

Journal: International Journal of Colorectal Disease

Authors: Aristotelis Nikitaras, Manousos-Georgios Pramateftakis, Konstantinos Perivoliotis, Sandra Maria Tsoti, Prokopis Christodoulou, Orestis Ioannidis, George Tzovaras

Corresponding author: Aristotelis Nikitaras, 1st Department of Surgery, Asklepieio General Hospital of Voula, Athens, Greece

Email: nikitaras.aristotelis@gmail.com

Online Resource 3: Forest plots for all outcomes- Overall complications forest plot

Effect size of each study
Estimated overall effect size
Estimated overall confidence interval
Confidence interval of effect size
Overall effect size value

ID	OR	Lower	Upper	p-value	Weight	Weight (%)
M. Wongyingsinn et al.	1.61	0.41	6.30	0.50	2.06	9.66
P. Lidder et al.	0.68	0.25	1.87	0.46	3.80	17.86
J. Webster et al.	0.45	0.07	2.76	0.39	1.17	5.49
S. M. Kumar et al.	0.35	0.12	1.07	0.07	3.12	14.65
Y. Deng et al.	1.37	0.29	6.53	0.69	1.57	7.39
N. Rizvanovi et al. 2023	0.26	0.08	0.87	0.03	2.66	12.49
S. E. Noblett et al.	1.00	0.06	18.08	1.00	0.46	2.15
N. Karimian et al.	0.55	0.10	2.89	0.48	1.38	6.48
M. Kaška et al.	0.83	0.24	2.86	0.77	2.53	11.86
H. Hamamoto et al.	2.29	0.67	7.82	0.19	2.55	11.97
Overall	0.72	0.47	1.11	0.13		

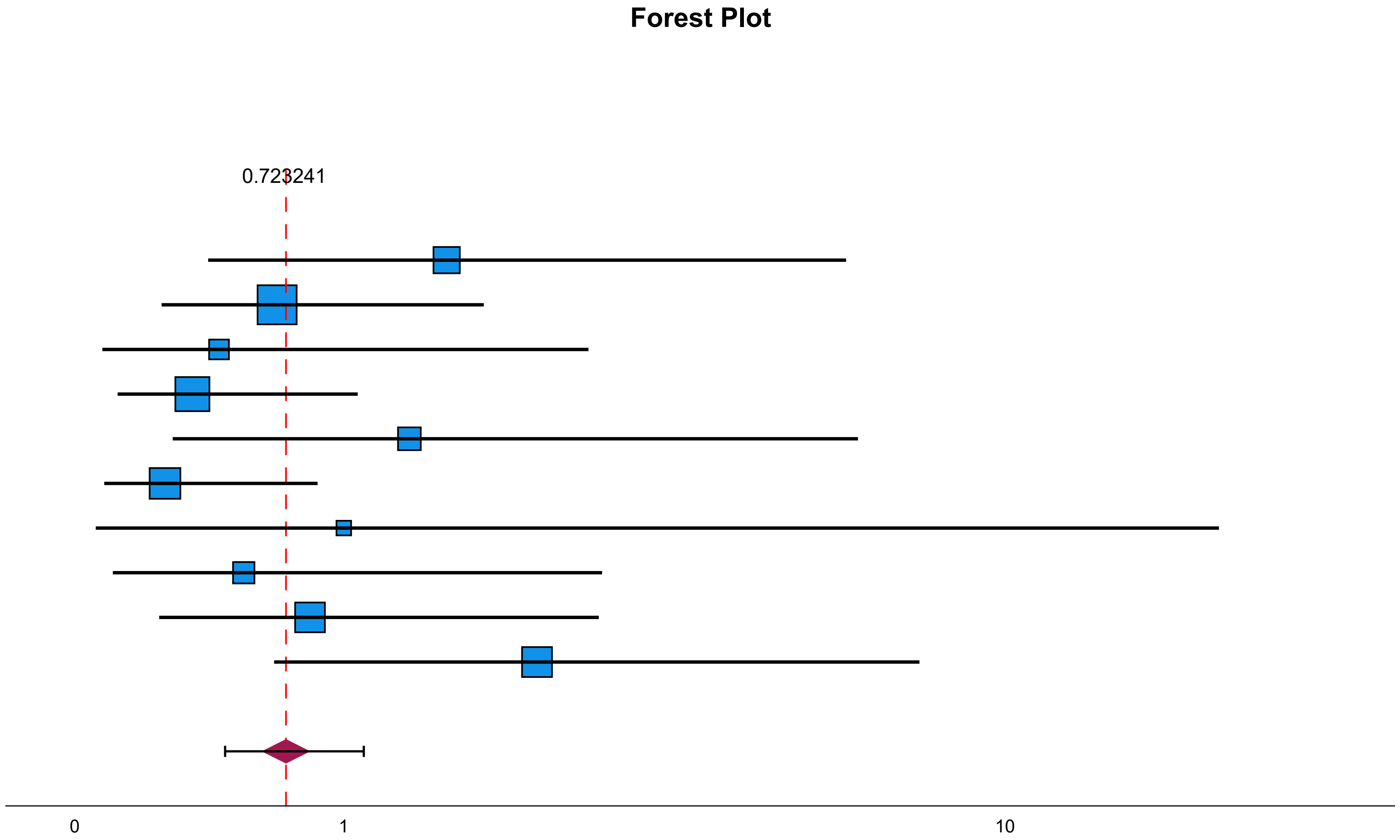

Model: Fixed-effects model
Heterogeneity: H-squared = 1.13, I-squared = 0.12
Homogeneity: Q = 10.17, df = 9, p-value = 0.34
Test of overall effect size: z = -1.50, p-value = 0.13
Axis is shown using log scale

Article title: Preoperative carbohydrate loading in elective colorectal surgery: postoperative complications and outcomes, a systematic review and meta-analysis

Journal: International Journal of Colorectal Disease

Authors: Aristotelis Nikitaras, Manousos-Georgios Pramateftakis, Konstantinos Perivoliotis, Sandra Maria Tsoti, Prokopis Christodoulou, Orestis Ioannidis, George Tzovaras

Corresponding author: Aristotelis Nikitaras, 1st Department of Surgery, Asklepieio General Hospital of Voula, Athens, Greece

Email: nikitaras.aristotelis@gmail.com

Online Resource 3: Forest plots for all outcomes - Septic complications forest plot

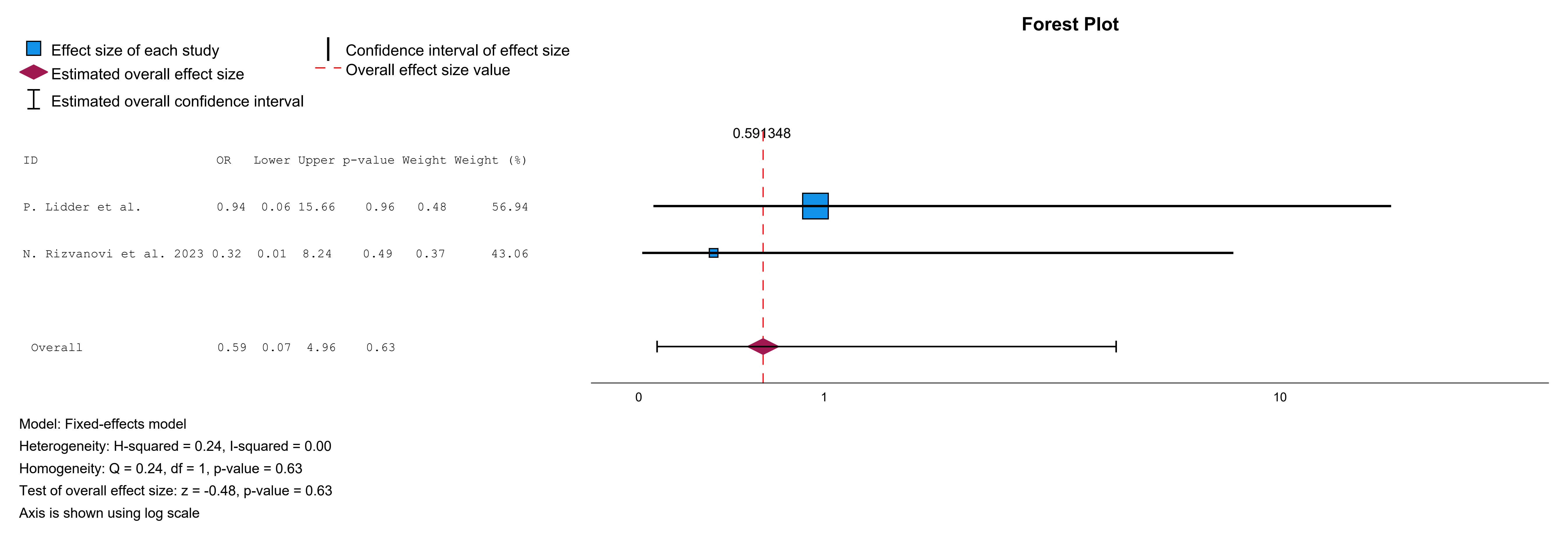
Forest Plot
Effect size of each study
Estimated overall effect size
Estimated overall confidence interval
Confidence interval of effect size
Overall effect size value
0.591348
ID OR Lower Upper p-value Weight Weight (%)
P. Lidder et al. 0.94 0.06 15.66 0.96 0.48 56.94
N. Rizvanovi et al. 2023 0.32 0.01 8.24 0.49 0.37 43.06
Overall 0.59 0.07 4.96 0.63
0
1
10
Model: Fixed-effects model
Heterogeneity: H-squared = 0.24, I-squared = 0.00
Homogeneity: Q = 0.24, df = 1, p-value = 0.63
Test of overall effect size: z = -0.48, p-value = 0.63
Axis is shown using log scale

Article title: Preoperative carbohydrate loading in elective colorectal surgery: postoperative complications and outcomes, a systematic review and meta-analysis

Journal: International Journal of Colorectal Disease

Authors: Aristotelis Nikitaras, Manousos-Georgios Pramateftakis, Konstantinos Perivoliotis, Sandra Maria Tsoti, Prokopis Christodoulou, Orestis Ioannidis, George Tzovaras

Corresponding author: Aristotelis Nikitaras, 1st Department of Surgery, Asklepieio General Hospital of Voula, Athens, Greece

Email: nikitaras.aristotelis@gmail.com

Online Resource 3: Forest plots for all outcomes - Ileus forest plot

Forest Plot

- Effect size of each study
- Estimated overall effect size
- Estimated overall confidence interval
- Confidence interval of effect size
- Overall effect size value

ID	OR	Lower	Upper	p-value	Weight	Weight (%)
M. Wongyingsinn et al.	2.06	0.18	23.88	0.56	0.64	19.44
P. Lidder et al.	2.00	0.34	11.82	0.44	1.22	36.96
N. Rizvanovi et al. 2023	0.32	0.01	8.24	0.49	0.37	11.11
N. Karimian et al.	3.44	0.13	91.79	0.46	0.36	10.82
H. Hamamoto et al.	3.43	0.34	34.86	0.30	0.71	21.68
Overall	1.96	0.67	5.77	0.22		

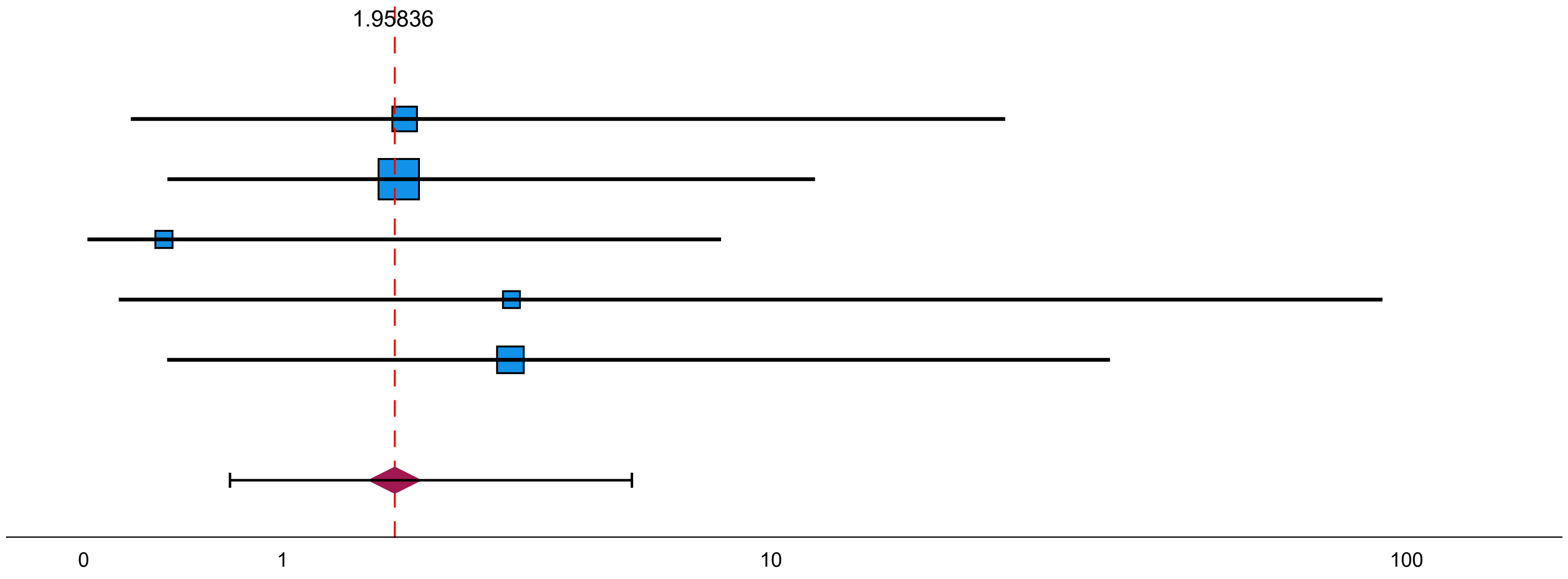

Model: Fixed-effects model

Heterogeneity: H-squared = 0.38, I-squared = 0.00

Homogeneity: Q = 1.53, df = 4, p-value = 0.82

Test of overall effect size: z = 1.22, p-value = 0.22

Axis is shown using log scale

Article title: Preoperative carbohydrate loading in elective colorectal surgery: postoperative complications and outcomes, a systematic review and meta-analysis

Journal: International Journal of Colorectal Disease

Authors: Aristotelis Nikitaras, Manousos-Georgios Pramateftakis, Konstantinos Perivoliotis, Sandra Maria Tsoti, Prokopis Christodoulou, Orestis Ioannidis, George Tzovaras

Corresponding author: Aristotelis Nikitaras, 1st Department of Surgery, Asklepieio General Hospital of Voula, Athens, Greece

Email: nikitaras.aristotelis@gmail.com

Online Resource 3: Forest plots for all outcomes - SSI forest plot

Effect size of each study
Estimated overall effect size
Estimated overall confidence interval
Confidence interval of effect size
Overall effect size value

ID	OR	Lower	Upper	p-value	Weight	Weight (%)
M. Wongyingsinn et al.	1.00	0.13	7.54	1.00	0.94	13.42
P. Lidder et al.	1.15	0.31	4.27	0.83	2.25	32.04
N. Rizvanovi et al. 2023	0.19	0.01	4.06	0.29	0.41	5.78
N. Karimian et al.	0.50	0.04	6.22	0.59	0.60	8.62
M. Kaška et al.	0.67	0.11	4.11	0.66	1.16	16.56
H. Hamamoto et al.	1.92	0.42	8.83	0.40	1.65	23.57
Overall	0.98	0.47	2.05	0.95		

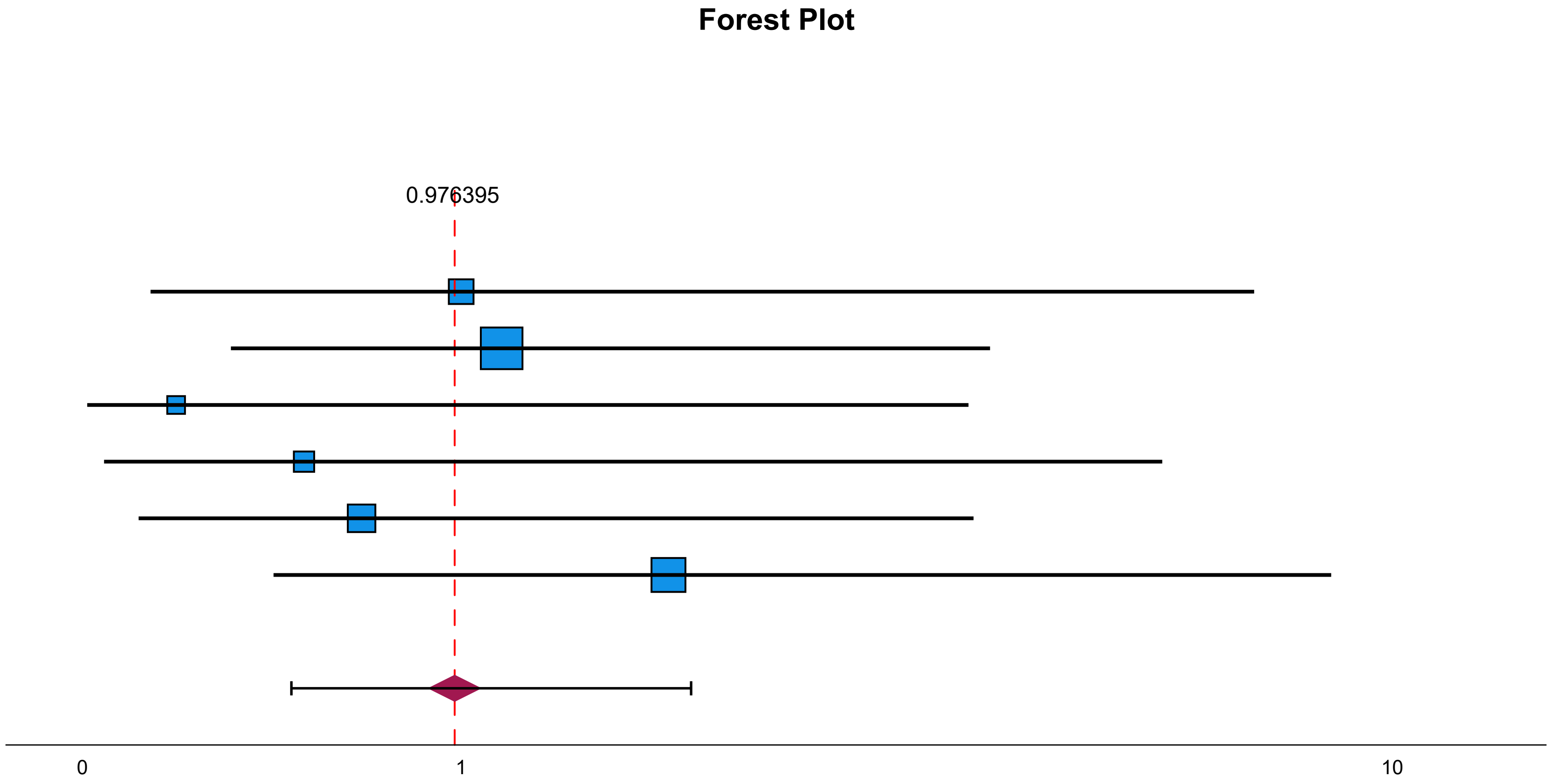

Model: Fixed-effects model
Heterogeneity: H-squared = 0.47, I-squared = 0.00
Homogeneity: Q = 2.37, df = 5, p-value = 0.80
Test of overall effect size: z = -0.06, p-value = 0.95
Axis is shown using log scale

Article title: Preoperative carbohydrate loading in elective colorectal surgery: postoperative complications and outcomes, a systematic review and meta-analysis

Journal: International Journal of Colorectal Disease

Authors: Aristotelis Nikitaras, Manousos-Georgios Pramateftakis, Konstantinos Perivoliotis, Sandra Maria Tsoti, Prokopis Christodoulou, Orestis Ioannidis, George Tzovaras

Corresponding author: Aristotelis Nikitaras, 1st Department of Surgery, Asklepieio General Hospital of Voula, Athens, Greece

Email: nikitaras.aristotelis@gmail.com

Online Resource 3: Forest plots for all outcomes - Pneumonia forest plot

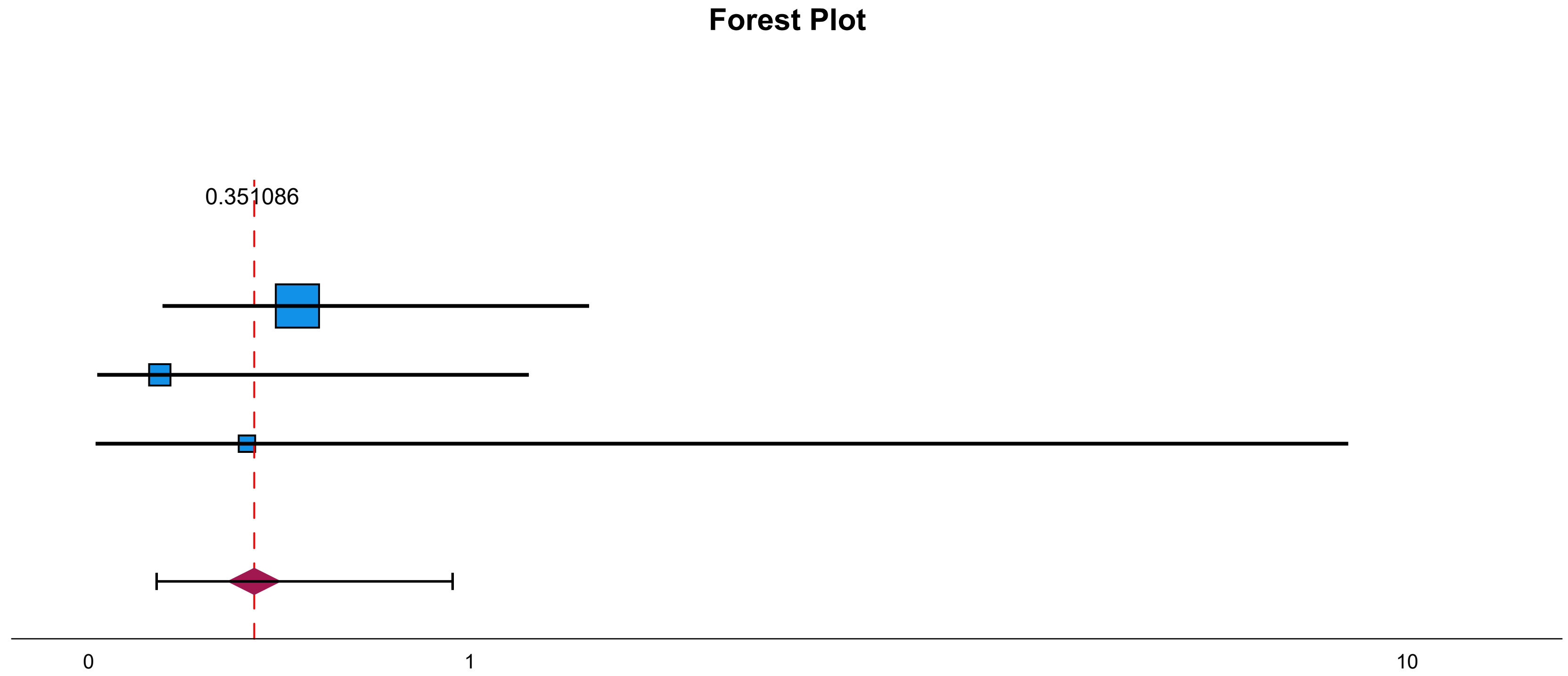

ID	OR	Lower	Upper	p-value	Weight	Weight (%)
P. Lidder et al.	0.46	0.14	1.48	0.19	2.82	70.80
N. Rizvanovi et al. 2023	0.14	0.02	1.23	0.08	0.80	20.23
N. Karimian et al.	0.33	0.01	8.88	0.51	0.36	8.97
Overall	0.35	0.13	0.94	0.04		

Model: Fixed-effects model

Heterogeneity: H-squared = 0.46, I-squared = 0.00

Homogeneity: Q = 0.91, df = 2, p-value = 0.63

Test of overall effect size: z = -2.09, p-value = 0.04

Axis is shown using log scale

Article title: Preoperative carbohydrate loading in elective colorectal surgery: postoperative complications and outcomes, a systematic review and meta-analysis

Journal: International Journal of Colorectal Disease

Authors: Aristotelis Nikitaras, Manousos-Georgios Pramateftakis, Konstantinos Perivoliotis, Sandra Maria Tsoti, Prokopis Christodoulou, Orestis Ioannidis, George Tzovaras

Corresponding author: Aristotelis Nikitaras, 1st Department of Surgery, Asklepieio General Hospital of Voula, Athens, Greece

Email: nikitaras.aristotelis@gmail.com

Online Resource 3: Forest plots for all outcomes - Cardiac complications forest plot

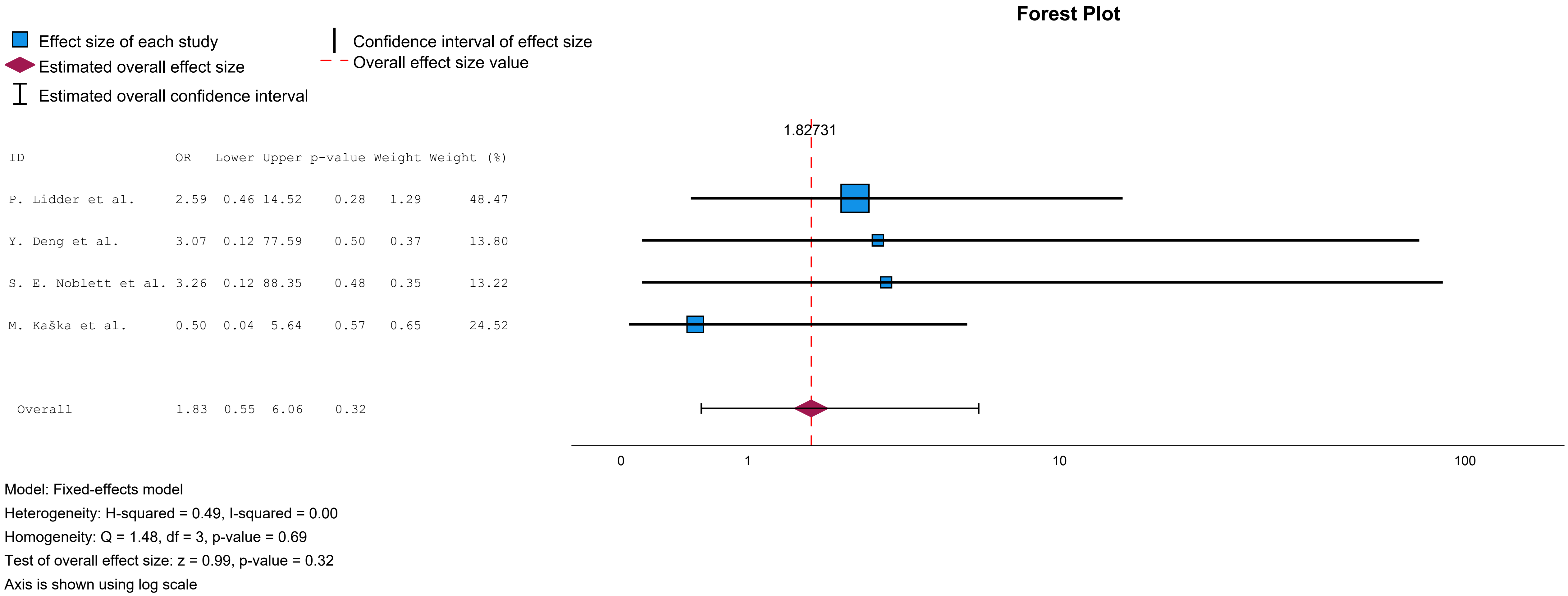

Forest Plot
Effect size of each study
Estimated overall effect size
Estimated overall confidence interval
Confidence interval of effect size
Overall effect size value
1.82731
ID OR Lower Upper p-value Weight Weight (%)
P. Lidder et al. 2.59 0.46 14.52 0.28 1.29 48.47
Y. Deng et al. 3.07 0.12 77.59 0.50 0.37 13.80
S. E. Noblett et al. 3.26 0.12 88.35 0.48 0.35 13.22
M. Kaška et al. 0.50 0.04 5.64 0.57 0.65 24.52
Overall 1.83 0.55 6.06 0.32
0
1
10
100
Model: Fixed-effects model
Heterogeneity: H-squared = 0.49, I-squared = 0.00
Homogeneity: Q = 1.48, df = 3, p-value = 0.69
Test of overall effect size: z = 0.99, p-value = 0.32
Axis is shown using log scale

Article title: Preoperative carbohydrate loading in elective colorectal surgery: postoperative complications and outcomes, a systematic review and meta-analysis

Journal: International Journal of Colorectal Disease

Authors: Aristotelis Nikitaras, Manousos-Georgios Pramateftakis, Konstantinos Perivoliotis, Sandra Maria Tsoti, Prokopis Christodoulou, Orestis Ioannidis, George Tzovaras

Corresponding author: Aristotelis Nikitaras, 1st Department of Surgery, Asklepieio General Hospital of Voula, Athens, Greece

Email: nikitaras.aristotelis@gmail.com

Online Resource 3: Forest plots for all outcomes - Thromboembolic complications forest plot

Effect size of each study
Estimated overall effect size
Estimated overall confidence interval
Confidence interval of effect size
Overall effect size value

ID	OR	Lower	Upper	p-value	Weight	Weight (%)
P. Lidder et al.	0.18	0.01	3.81	0.27	0.41	28.44
N. Rizvanovi et al. 2023	0.32	0.01	8.24	0.49	0.37	25.65
M. Kaška et al.	0.50	0.04	5.64	0.57	0.65	45.91
Overall	0.33	0.06	1.71	0.19		

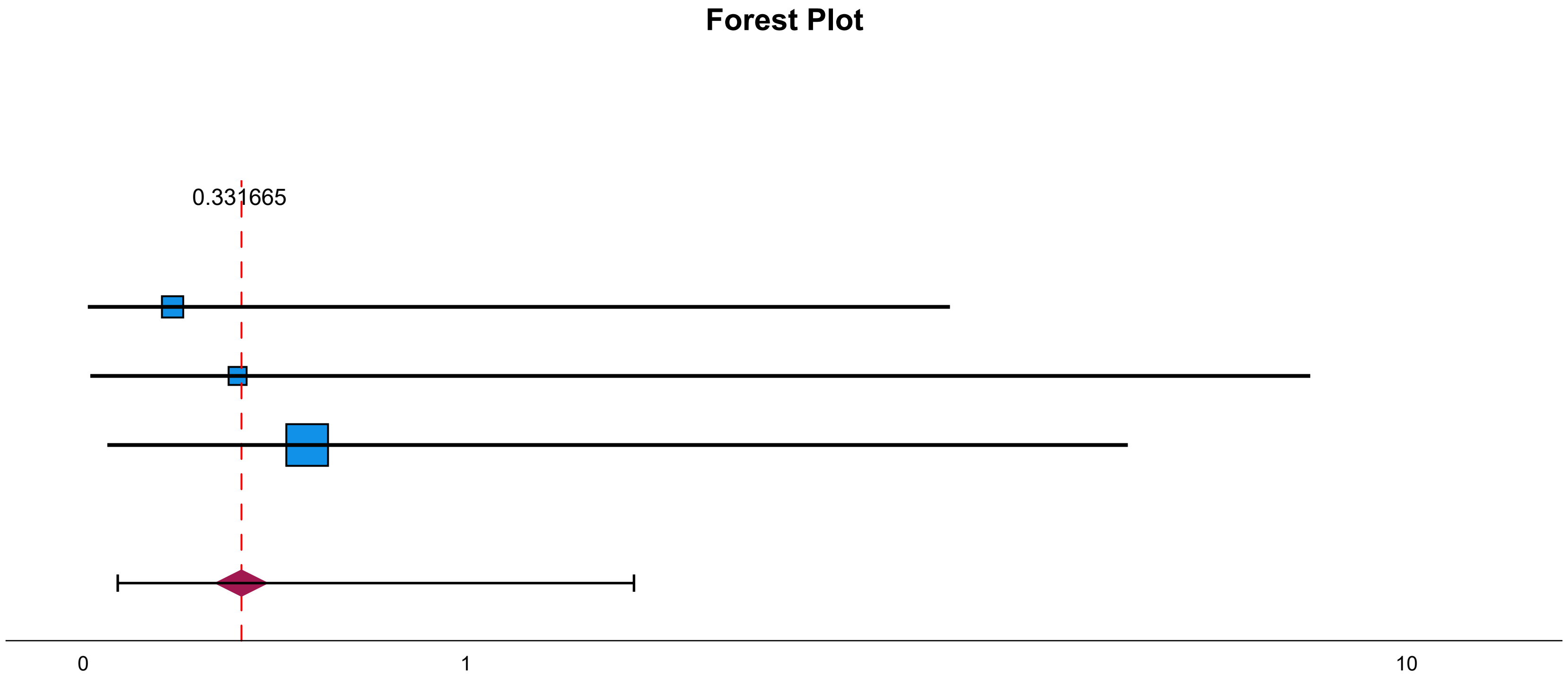

Model: Fixed-effects model
Heterogeneity: H-squared = 0.14, I-squared = 0.00
Homogeneity: Q = 0.28, df = 2, p-value = 0.87
Test of overall effect size: z = -1.32, p-value = 0.19
Axis is shown using log scale

Article title: Preoperative carbohydrate loading in elective colorectal surgery: postoperative complications and outcomes, a systematic review and meta-analysis

Journal: International Journal of Colorectal Disease

Authors: Aristotelis Nikitaras, Manousos-Georgios Pramateftakis, Konstantinos Perivoliotis, Sandra Maria Tsoti, Prokopis Christodoulou, Orestis Ioannidis, George Tzovaras

Corresponding author: Aristotelis Nikitaras, 1st Department of Surgery, Asklepieio General Hospital of Voula, Athens, Greece

Email: nikitaras.aristotelis@gmail.com

Online Resource 3: Forest plots for all outcomes - First flatus forest plot

Effect size of each study
Estimated overall effect size
No-effect value
Confidence interval of effect size
Overall effect size value
Estimated overall confidence interva

Forest Plot

ID	Mean difference	Std. Error	Lower	Upper	p-value	Weight	Weight (%)
N. Rizvanovi et al. ...	-0.60	0.14	-0.88	-0.32	0.00	50.00	29.88
J. Webster et al.	-0.63	0.10	-0.83	-0.43	0.00	96.07	57.42
N. Rizvanovi et al. 2023	-0.11	0.28	-0.66	0.44	0.69	12.82	7.66
H. Hamamoto et al.	-1.00	0.34	-1.67	-0.33	0.00	8.43	5.04
Overall	-0.60	0.08	-0.75	-0.45	0.00		

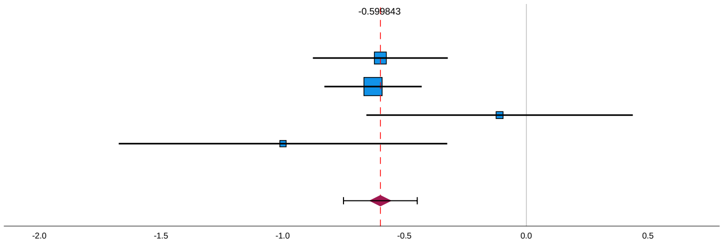

Model: Fixed-effects model
Heterogeneity: H-squared = 1.50, I-squared = 0.34
Homogeneity: Q = 4.51, df = 3, p-value = 0.21
Test of overall effect size: z = -7.76, p-value = 0.00

Article title: Preoperative carbohydrate loading in elective colorectal surgery: postoperative complications and outcomes, a systematic review and meta-analysis

Journal: International Journal of Colorectal Disease

Authors: Aristotelis Nikitaras, Manousos-Georgios Pramateftakis, Konstantinos Perivoliotis, Sandra Maria Tsoti, Prokopis Christodoulou, Orestis Ioannidis, George Tzovaras

Corresponding author: Aristotelis Nikitaras, 1st Department of Surgery, Asklepieio General Hospital of Voula, Athens, Greece

Email: nikitaras.aristotelis@gmail.com

Online Resource 3: Forest plots for all outcomes - First defecation forest plot

Forest Plot

- Effect size of each study
- Estimated overall effect size
- No-effect value
- Confidence interval of effect size
- Overall effect size value
- Estimated overall confidence interva

ID	Mean difference	Std. Error	Lower	Upper	p-value	Weight	Weight (%)
N. Rizvanovi et al. ...	-0.80	0.20	-1.19	-0.41	0.00	5.20	23.92
J. Webster et al.	-0.92	0.12	-1.15	-0.69	0.00	5.96	27.45
S. M. Kumar et al.	-0.11	0.20	-0.50	0.28	0.58	5.16	23.73
N. Rizvanovi et al. 2023	0.23	0.49	-0.74	1.20	0.64	2.51	11.57
H. Hamamoto et al.	-1.00	0.44	-1.86	-0.14	0.02	2.90	13.33
Overall	-0.58	0.21	-1.00	-0.16	0.01		

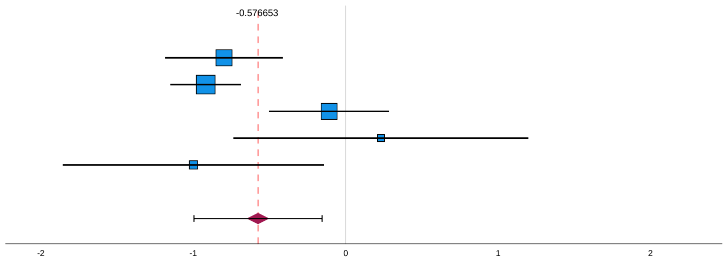

Model: Random-effects model

Heterogeneity: Tau-squared = 0.15, H-squared = 4.16, I-squared = 0.76

Homogeneity: Q = 16.34, df = 4, p-value = 0.00

Test of overall effect size: z = -2.69, p-value = 0.01

Article title: Preoperative carbohydrate loading in elective colorectal surgery: postoperative complications and outcomes, a systematic review and meta-analysis

Journal: International Journal of Colorectal Disease

Authors: Aristotelis Nikitaras, Manousos-Georgios Pramateftakis, Konstantinos Perivoliotis, Sandra Maria Tsoti, Prokopis Christodoulou, Orestis Ioannidis, George Tzovaras

Corresponding author: Aristotelis Nikitaras, 1st Department of Surgery, Asklepieio General Hospital of Voula, Athens, Greece

Email: nikitaras.aristotelis@gmail.com

Online Resource 3: Forest plots for all outcomes - Mobilisation forest plot

Effect size of each study
Estimated overall effect size
No-effect value
Confidence interval of effect size
Overall effect size value
Estimated overall confidence interva

ID	Mean difference	Std. Error	Lower	Upper	p-value	Weight	Weight (%)
N. Rizvanovi et al. ...	-0.60	0.13	-0.85	-0.35	0.00	60.98	64.02
S. M. Kumar et al.	-0.50	0.38	-1.24	0.24	0.19	6.92	7.27
Y. Deng et al.	-0.25	0.19	-0.62	0.12	0.19	27.34	28.71
Overall	-0.49	0.10	-0.69	-0.29	0.00		

Forest Plot

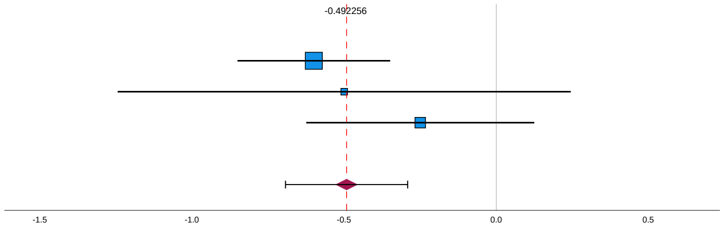

Model: Fixed-effects model
Heterogeneity: H-squared = 1.16, I-squared = 0.14
Homogeneity: Q = 2.31, df = 2, p-value = 0.31
Test of overall effect size: z = -4.80, p-value = 0.00

Article title: Preoperative carbohydrate loading in elective colorectal surgery: postoperative complications and outcomes, a systematic review and meta-analysis

Journal: International Journal of Colorectal Disease

Authors: Aristotelis Nikitaras, Manousos-Georgios Pramateftakis, Konstantinos Perivoliotis, Sandra Maria Tsoti, Prokopis Christodoulou, Orestis Ioannidis, George Tzovaras

Corresponding author: Aristotelis Nikitaras, 1st Department of Surgery, Asklepieio General Hospital of Voula, Athens, Greece

Email: nikitaras.aristotelis@gmail.com

Online Resource 3: Forest plots for all outcomes - LOS forest plot

Effect size of each study
Estimated overall effect size
No-effect value
Confidence interval of effect size
Overall effect size value
Estimated overall confidence interva

ID	Mean difference	Std. Error	Lower	Upper	p-value	Weight	Weight (%)
N. Rizvanovi et al. ...	-1.10	0.23	-1.56	-0.64	0.00	2.20	20.80
P. Lidder et al.	-1.50	1.13	-3.71	0.71	0.18	0.60	5.64
J. Webster et al.	-0.20	0.18	-0.55	0.15	0.27	2.32	21.88
S. M. Kumar et al.	-1.60	0.24	-2.07	-1.13	0.00	2.19	20.66
Y. Deng et al.	0.00	0.57	-1.12	1.12	1.00	1.38	13.06
N. Rizvanovi et al. 2023	-2.25	0.76	-3.75	-0.75	0.00	1.02	9.60
N. Karimian et al.	-1.00	0.88	-2.72	0.72	0.26	0.85	8.05
H. Hamamoto et al.	0.00	5.57	-10.91	10.91	1.00	0.03	0.30
Overall	-0.98	0.31	-1.59	-0.38	0.00		

Forest Plot

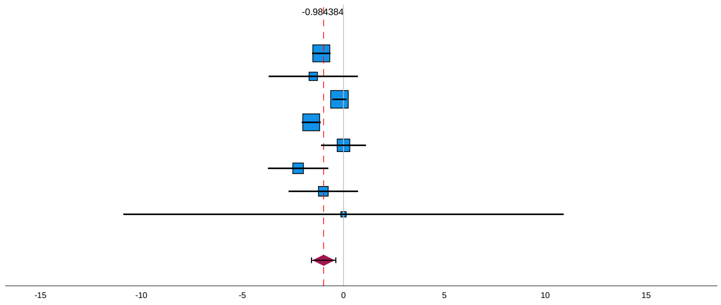

Model: Random-effects model
Heterogeneity: Tau-squared = 0.40, H-squared = 3.94, I-squared = 0.75
Homogeneity: Q = 29.87, df = 7, p-value = 0.00
Test of overall effect size: z = -3.20, p-value = 0.00
