# Supplementary material for: Preoperative carbohydrate loading in elective colorectal surgery: postoperative complications and outcomes, a systematic review and meta-analysis
Source: Int J Colorectal Dis. 2026 Apr 18;41(1):95. doi: 10.1007/s00384-026-05125-7 (PMC13222900; doi:10.1007/s00384-026-05125-7)
Supplement: Supplementary file 4 — Supplementary file4 Funnel plots for all outcomes (PDF 1.12 MB) [file 384_2026_5125_MOESM4_ESM.pdf]

Article title: Preoperative carbohydrate loading in elective colorectal surgery: postoperative complications and outcomes, a systematic review and meta-analysis

Journal: International Journal of Colorectal Disease

Authors: Aristotelis Nikitaras, Manousos-Georgios Pramateftakis, Konstantinos Perivoliotis, Sandra Maria Tsoti, Prokopis Christodoulou, Orestis Ioannidis, George Tzovaras

Corresponding author: Aristotelis Nikitaras, 1st Department of Surgery, Asklepieio General Hospital of Voula, Athens, Greece

Email: nikitaras.aristotelis@gmail.com

Online Resource 4: Funnel plots for all outcomes - Overall complications funnel plot

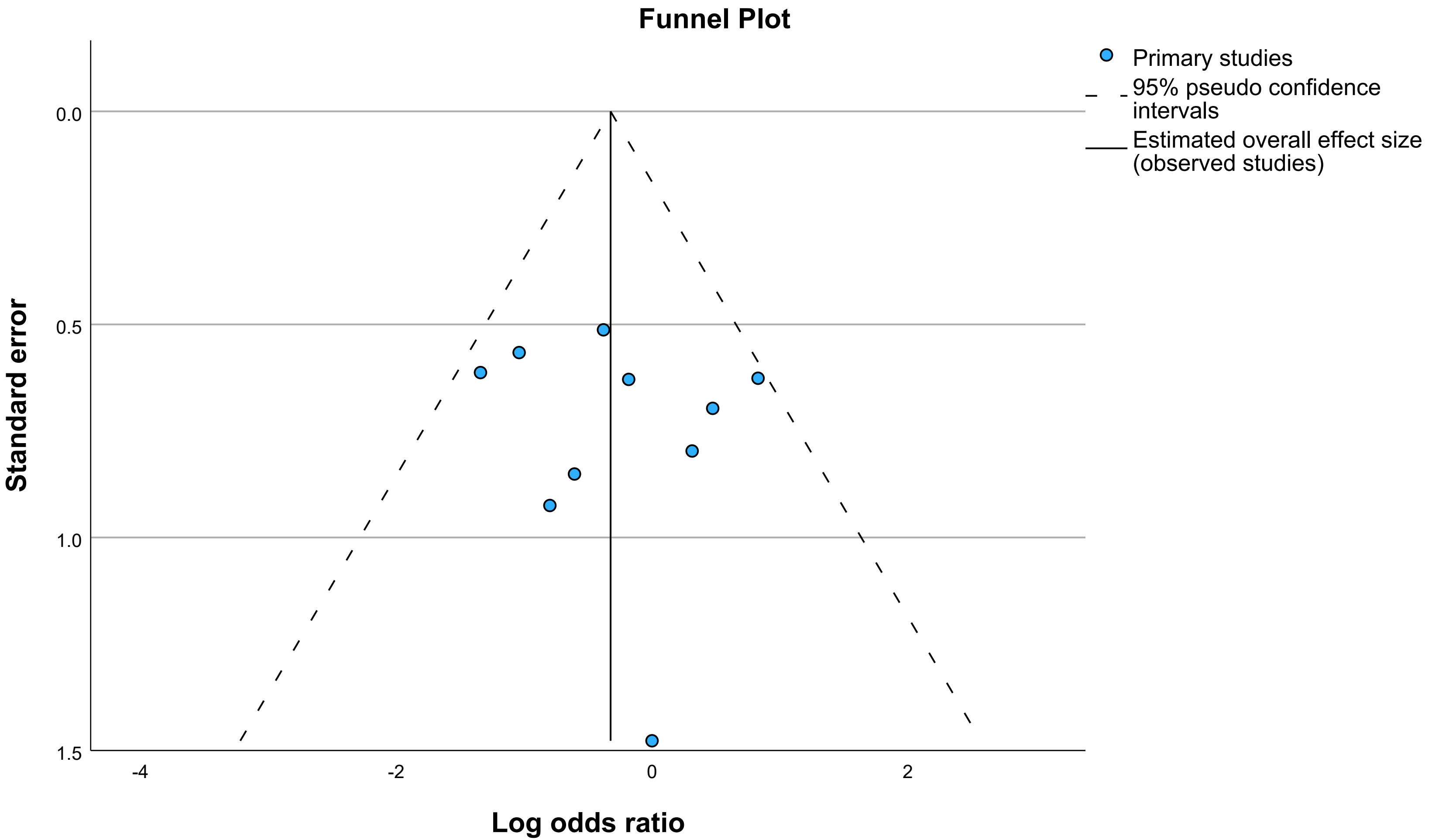
Funnel Plot
Primary studies
95% pseudo confidence intervals
Estimated overall effect size (observed studies)
Standard error
0.0
0.5
1.0
1.5
-4
-2
0
2
Log odds ratio

Article title: Preoperative carbohydrate loading in elective colorectal surgery: postoperative complications and outcomes, a systematic review and meta-analysis

Journal: International Journal of Colorectal Disease

Authors: Aristotelis Nikitaras, Manousos-Georgios Pramateftakis, Konstantinos Perivoliotis, Sandra Maria Tsoti, Prokopis Christodoulou, Orestis Ioannidis, George Tzovaras

Corresponding author: Aristotelis Nikitaras, 1st Department of Surgery, Asklepieio General Hospital of Voula, Athens, Greece

Email: nikitaras.aristotelis@gmail.com

Online Resource 4: Funnel plots for all outcomes - Septic complications funnel plot

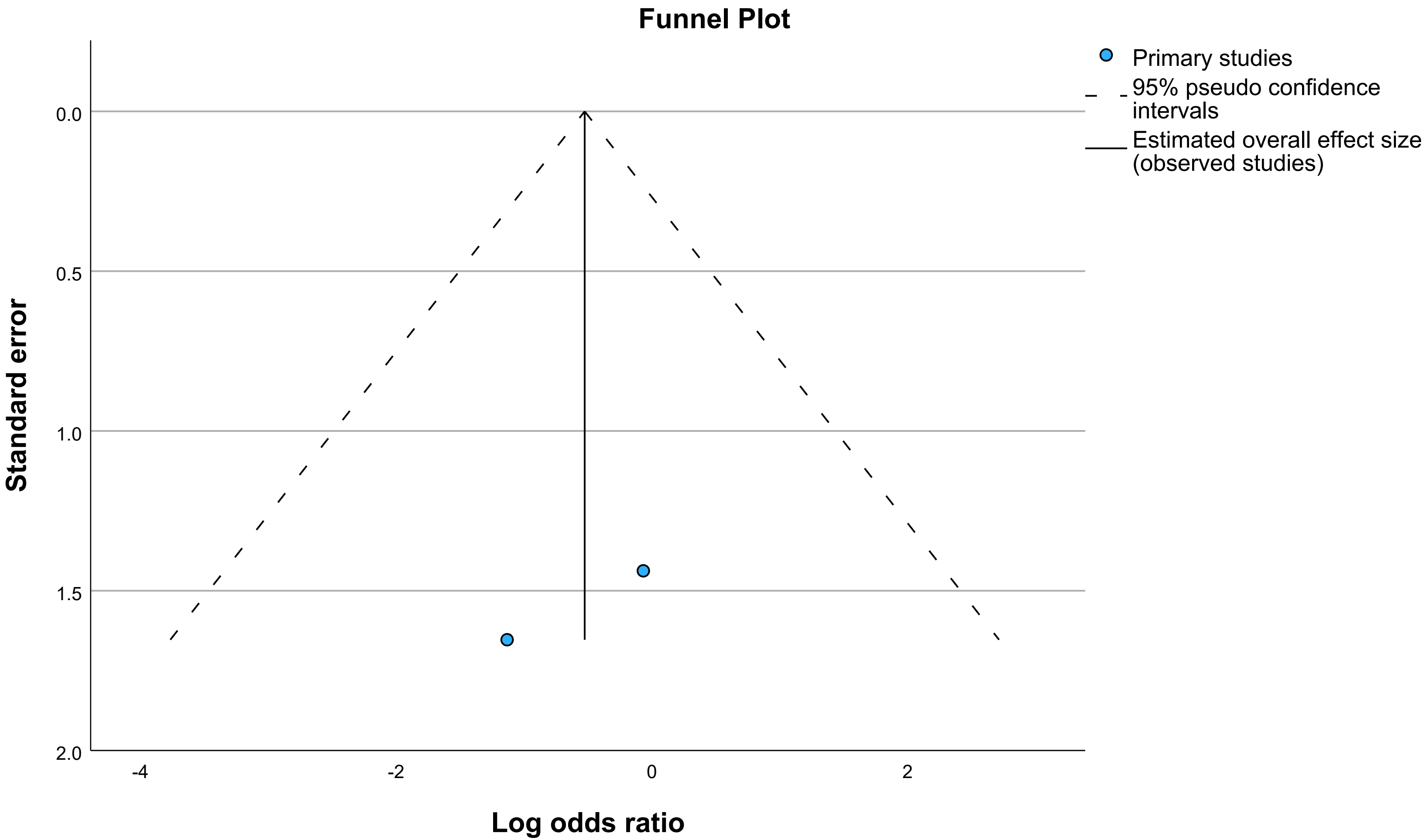

Funnel Plot
Primary studies
95% pseudo confidence intervals
Estimated overall effect size (observed studies)
Standard error
0.0
0.5
1.0
1.5
2.0
-4
-2
0
2
Log odds ratio

Article title: Preoperative carbohydrate loading in elective colorectal surgery: postoperative complications and outcomes, a systematic review and meta-analysis

Journal: International Journal of Colorectal Disease

Authors: Aristotelis Nikitaras, Manousos-Georgios Pramateftakis, Konstantinos Perivoliotis, Sandra Maria Tsoti, Prokopis Christodoulou, Orestis Ioannidis, George Tzovaras

Corresponding author: Aristotelis Nikitaras, 1st Department of Surgery, Asklepieio General Hospital of Voula, Athens, Greece

Email: nikitaras.aristotelis@gmail.com

Online Resource 4: Funnel plots for all outcomes - Ileus funnel plot

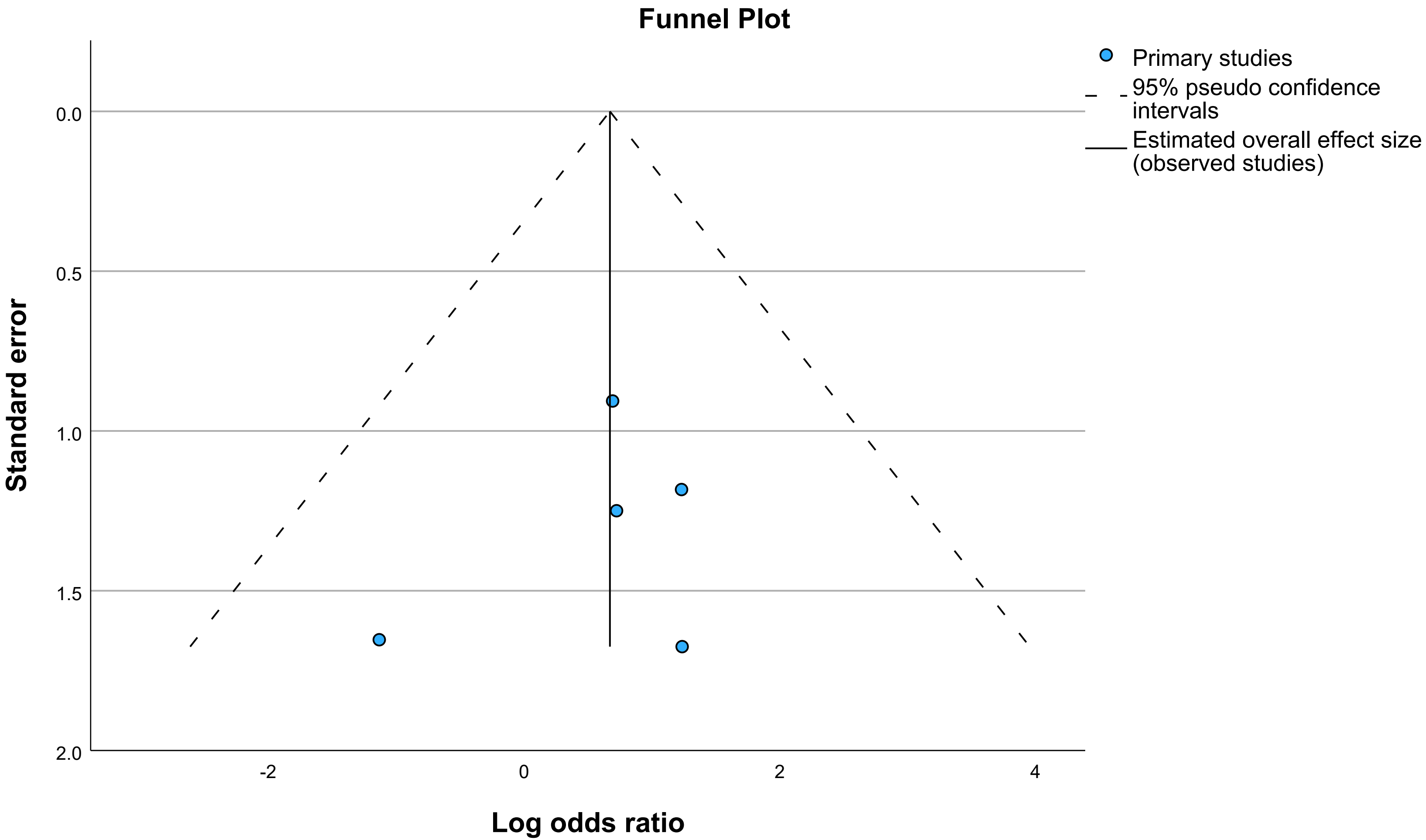

Funnel Plot
Primary studies
95% pseudo confidence intervals
Estimated overall effect size (observed studies)
Standard error
0.0
0.5
1.0
1.5
2.0
-2
0
2
4
Log odds ratio

Article title: Preoperative carbohydrate loading in elective colorectal surgery: postoperative complications and outcomes, a systematic review and meta-analysis

Journal: International Journal of Colorectal Disease

Authors: Aristotelis Nikitaras, Manousos-Georgios Pramateftakis, Konstantinos Perivoliotis, Sandra Maria Tsoti, Prokopis Christodoulou, Orestis Ioannidis, George Tzovaras

Corresponding author: Aristotelis Nikitaras, 1st Department of Surgery, Asklepieio General Hospital of Voula, Athens, Greece

Email: nikitaras.aristotelis@gmail.com

Online Resource 4: Funnel plots for all outcomes - SSI funnel plot

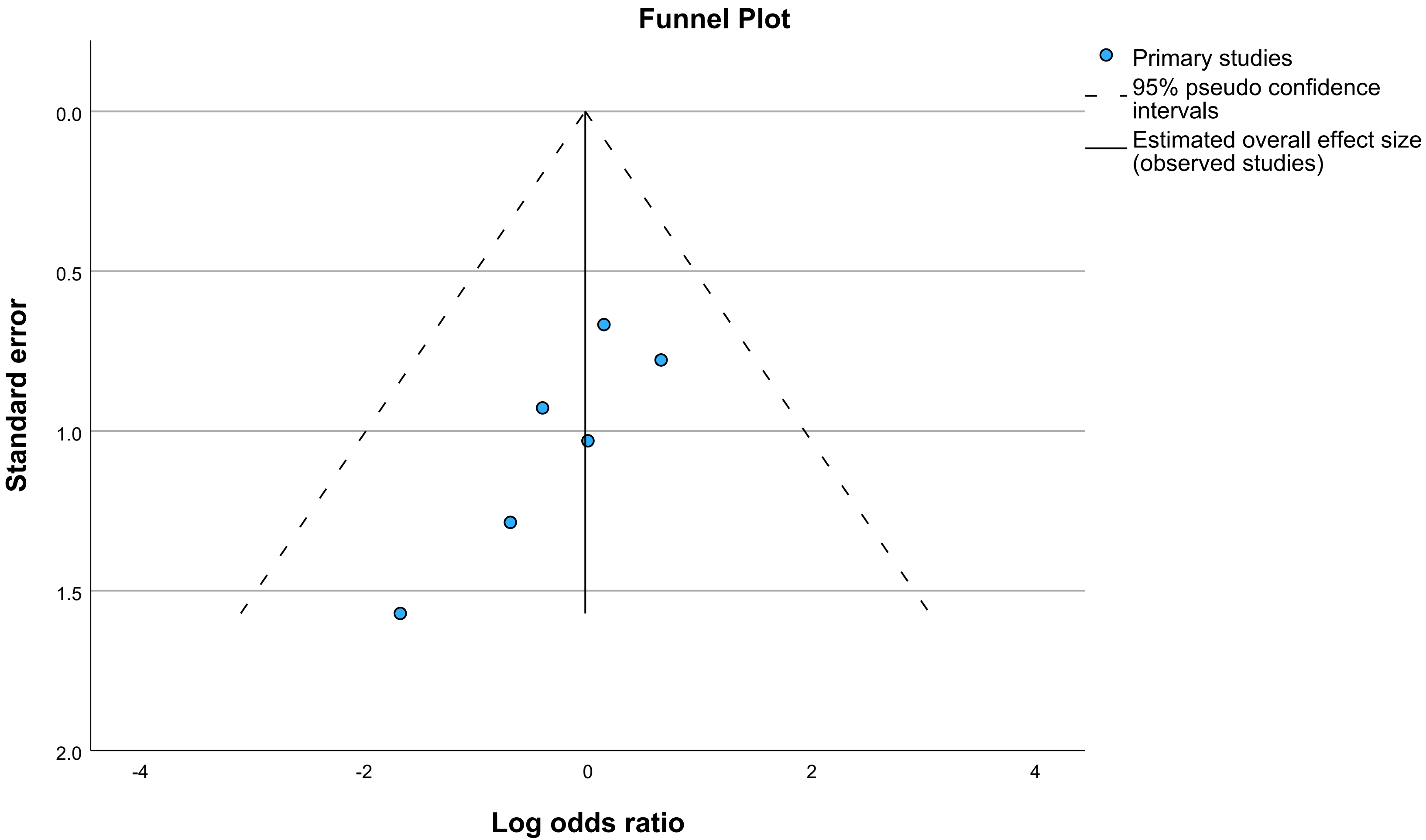

Funnel Plot
Primary studies
95% pseudo confidence intervals
Estimated overall effect size (observed studies)
Standard error
0.0
0.5
1.0
1.5
2.0
-4
-2
0
2
4
Log odds ratio

Article title: Preoperative carbohydrate loading in elective colorectal surgery: postoperative complications and outcomes, a systematic review and meta-analysis

Journal: International Journal of Colorectal Disease

Authors: Aristotelis Nikitaras, Manousos-Georgios Pramateftakis, Konstantinos Perivoliotis, Sandra Maria Tsoti, Prokopis Christodoulou, Orestis Ioannidis, George Tzovaras

Corresponding author: Aristotelis Nikitaras, 1st Department of Surgery, Asklepieio General Hospital of Voula, Athens, Greece

Email: nikitaras.aristotelis@gmail.com

Online Resource 4: Funnel plots for all outcomes - Pneumonia funnel plot

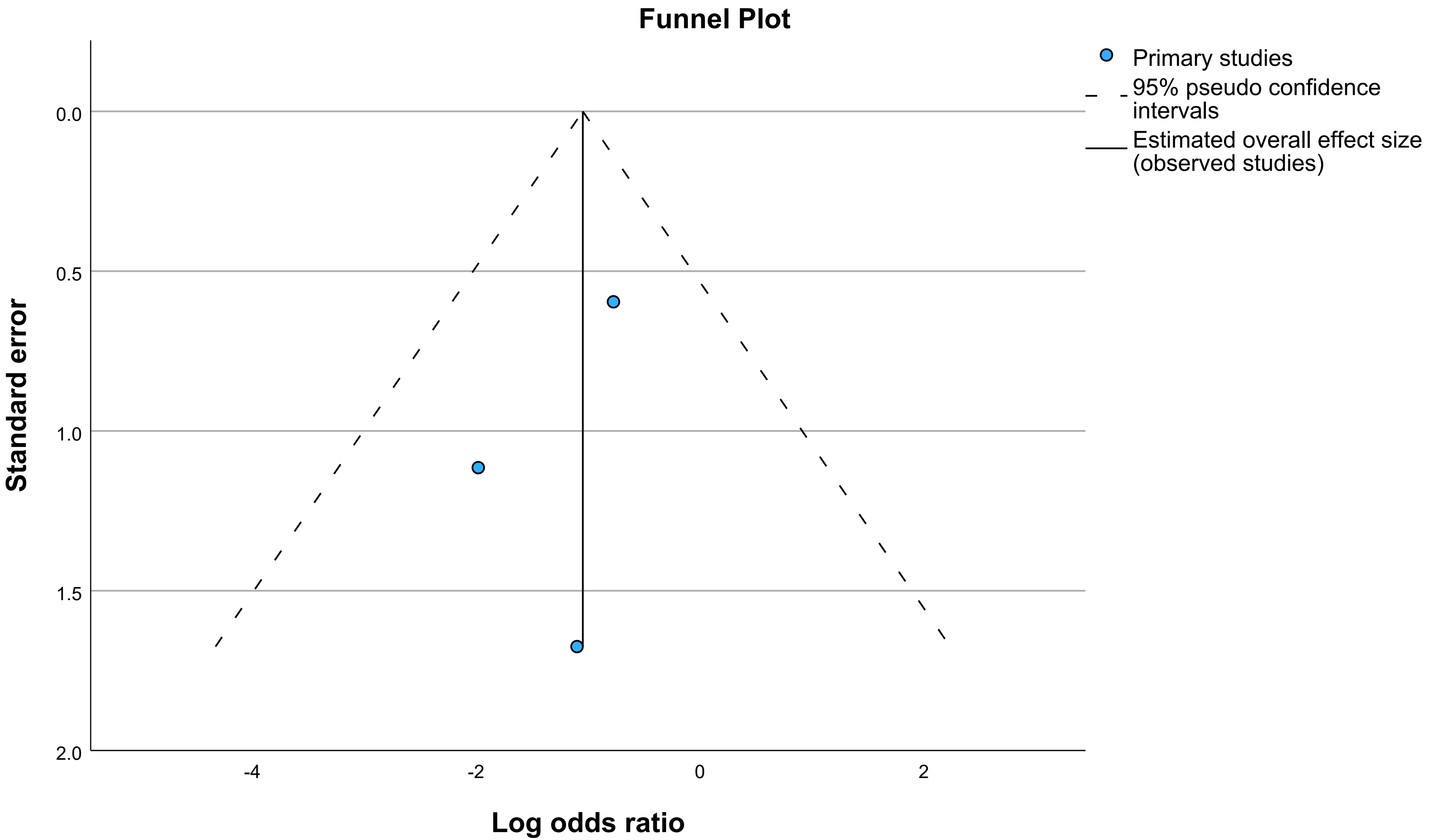
Funnel Plot
Primary studies
95% pseudo confidence intervals
Estimated overall effect size (observed studies)
Standard error
0.0
0.5
1.0
1.5
2.0
-4
-2
0
2
Log odds ratio

Article title: Preoperative carbohydrate loading in elective colorectal surgery: postoperative complications and outcomes, a systematic review and meta-analysis

Journal: International Journal of Colorectal Disease

Authors: Aristotelis Nikitaras, Manousos-Georgios Pramateftakis, Konstantinos Perivoliotis, Sandra Maria Tsoti, Prokopis Christodoulou, Orestis Ioannidis, George Tzovaras

Corresponding author: Aristotelis Nikitaras, 1st Department of Surgery, Asklepieio General Hospital of Voula, Athens, Greece

Email: nikitaras.aristotelis@gmail.com

Online Resource 4: Funnel plots for all outcomes – Thromboembolic complications funnel plot

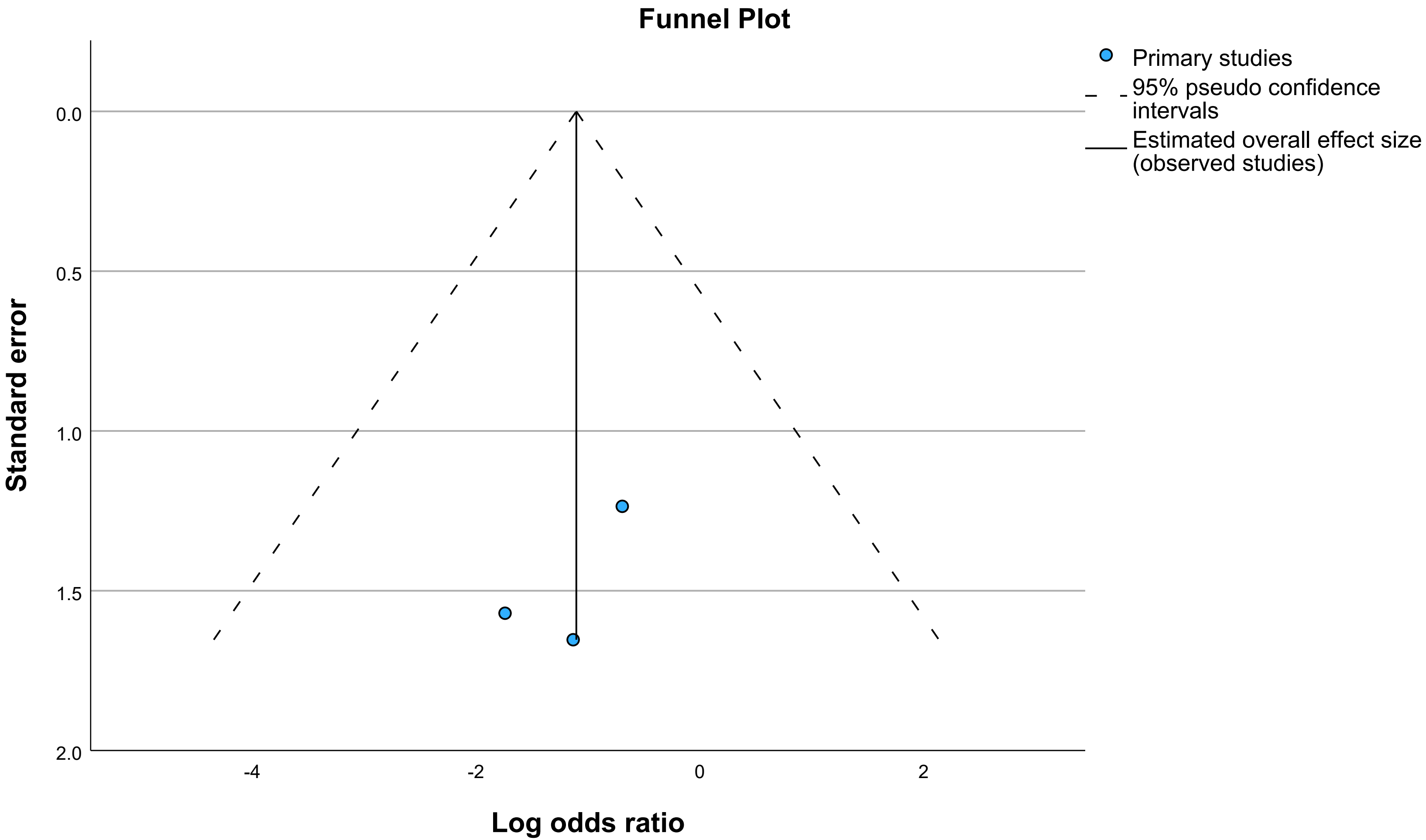
Funnel Plot
Primary studies
95% pseudo confidence intervals
Estimated overall effect size (observed studies)
Standard error
0.0
0.5
1.0
1.5
2.0
-4
-2
0
2
Log odds ratio

Article title: Preoperative carbohydrate loading in elective colorectal surgery: postoperative complications and outcomes, a systematic review and meta-analysis

Journal: International Journal of Colorectal Disease

Authors: Aristotelis Nikitaras, Manousos-Georgios Pramateftakis, Konstantinos Perivoliotis, Sandra Maria Tsoti, Prokopis Christodoulou, Orestis Ioannidis, George Tzovaras

Corresponding author: Aristotelis Nikitaras, 1st Department of Surgery, Asklepieio General Hospital of Voula, Athens, Greece

Email: nikitaras.aristotelis@gmail.com

Online Resource 4: Funnel plots for all outcomes – LOS funnel plot

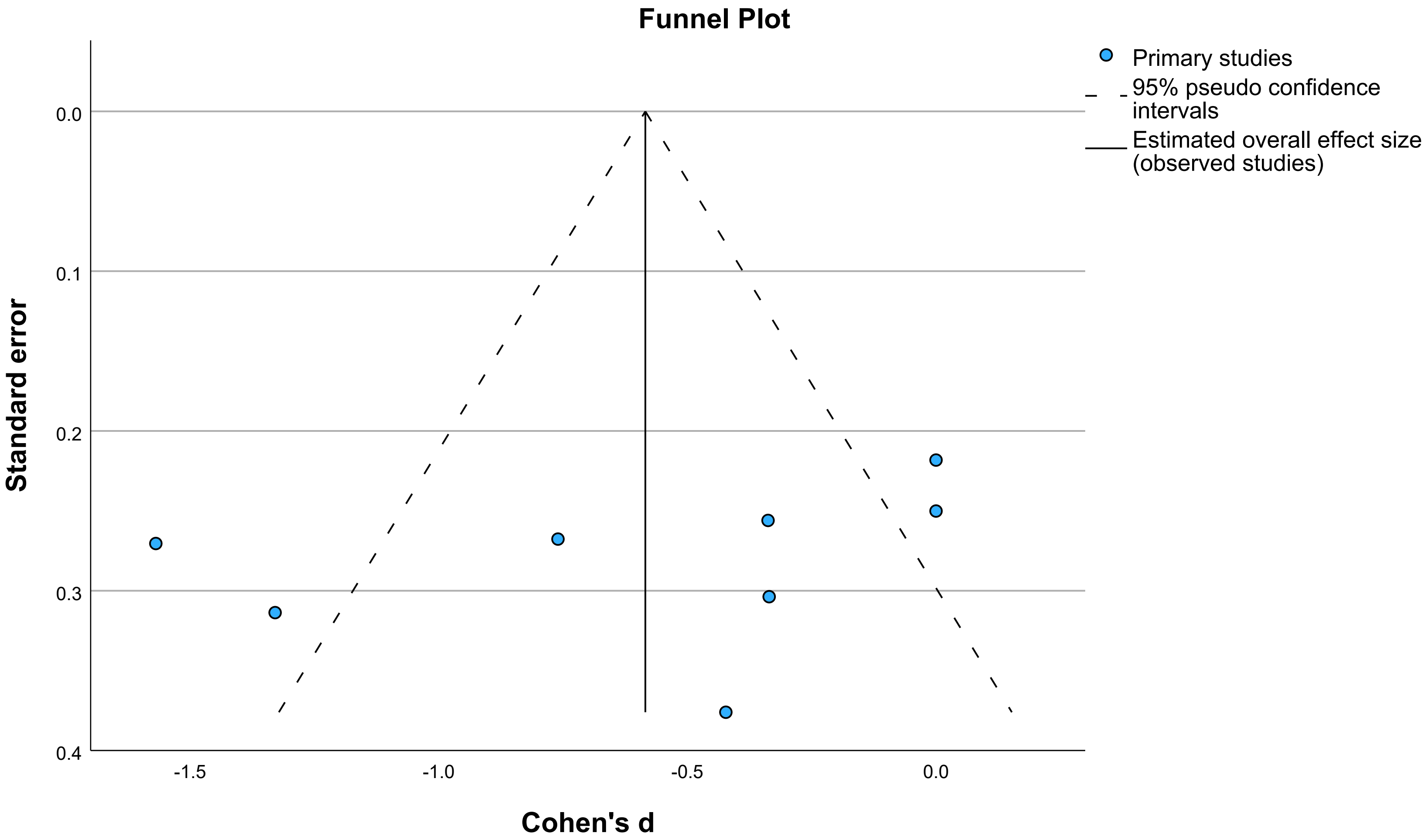
Funnel Plot
Primary studies
95% pseudo confidence intervals
Estimated overall effect size (observed studies)
Standard error
0.0
0.1
0.2
0.3
0.4
-1.5
-1.0
-0.5
0.0
Cohen's d

Article title: Preoperative carbohydrate loading in elective colorectal surgery: postoperative complications and outcomes, a systematic review and meta-analysis

Journal: International Journal of Colorectal Disease

Authors: Aristotelis Nikitaras, Manousos-Georgios Pramateftakis, Konstantinos Perivoliotis, Sandra Maria Tsoti, Prokopis Christodoulou, Orestis Ioannidis, George Tzovaras

Corresponding author: Aristotelis Nikitaras, 1st Department of Surgery, Asklepieio General Hospital of Voula, Athens, Greece

Email: nikitaras.aristotelis@gmail.com

Online Resource 4: Funnel plots for all outcomes – Anastomotic leak funnel plot

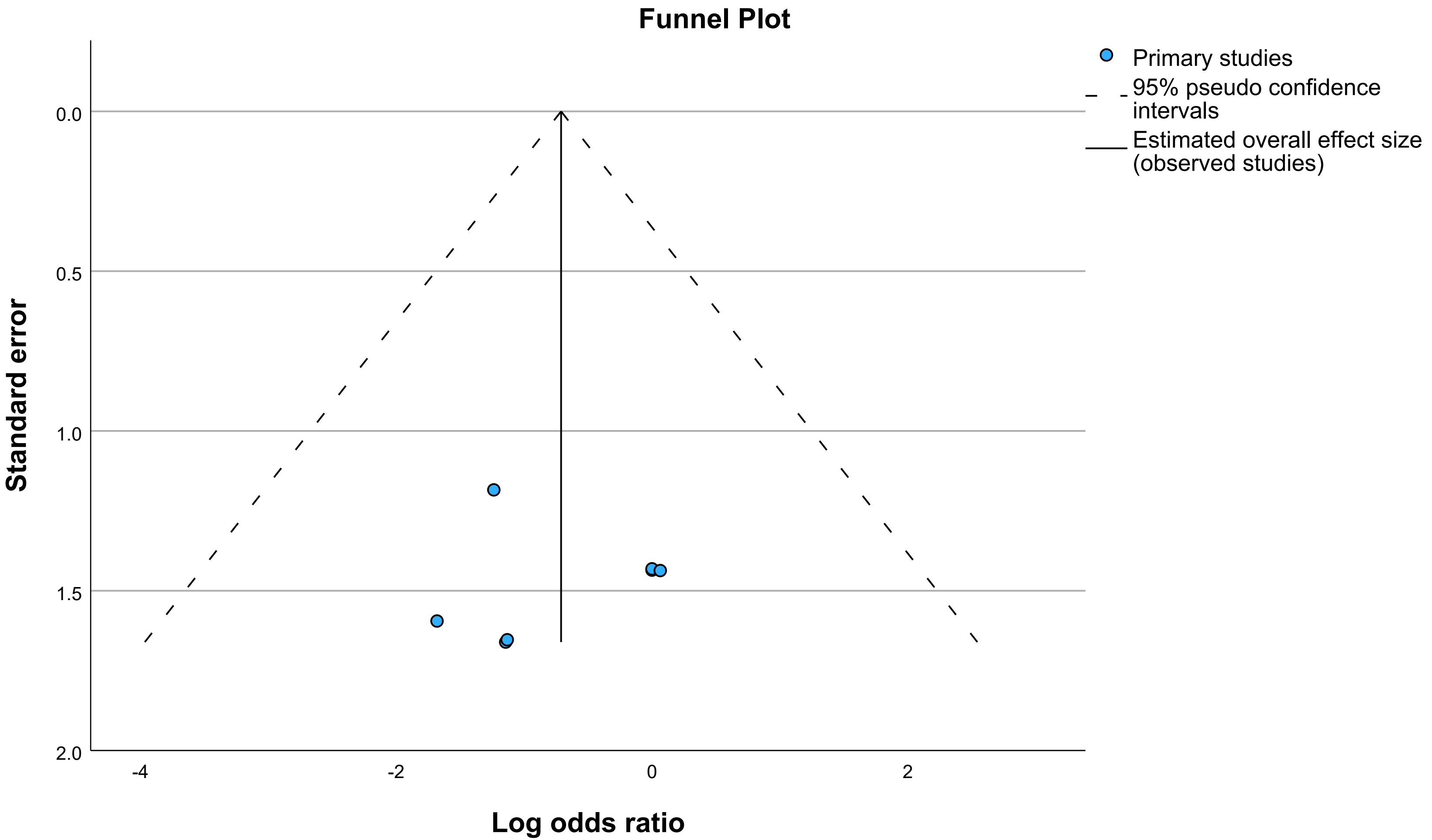
Funnel Plot
Primary studies
95% pseudo confidence intervals
Estimated overall effect size (observed studies)
Standard error
0.0
0.5
1.0
1.5
2.0
-4
-2
0
2
Log odds ratio

Article title: Preoperative carbohydrate loading in elective colorectal surgery: postoperative complications and outcomes, a systematic review and meta-analysis

Journal: International Journal of Colorectal Disease

Authors: Aristotelis Nikitaras, Manousos-Georgios Pramateftakis, Konstantinos Perivoliotis, Sandra Maria Tsoti, Prokopis Christodoulou, Orestis Ioannidis, George Tzovaras

Corresponding author: Aristotelis Nikitaras, 1st Department of Surgery, Asklepieio General Hospital of Voula, Athens, Greece

Email: nikitaras.aristotelis@gmail.com

Online Resource 4: Funnel plots for all outcomes – Cardiac complications funnel plot

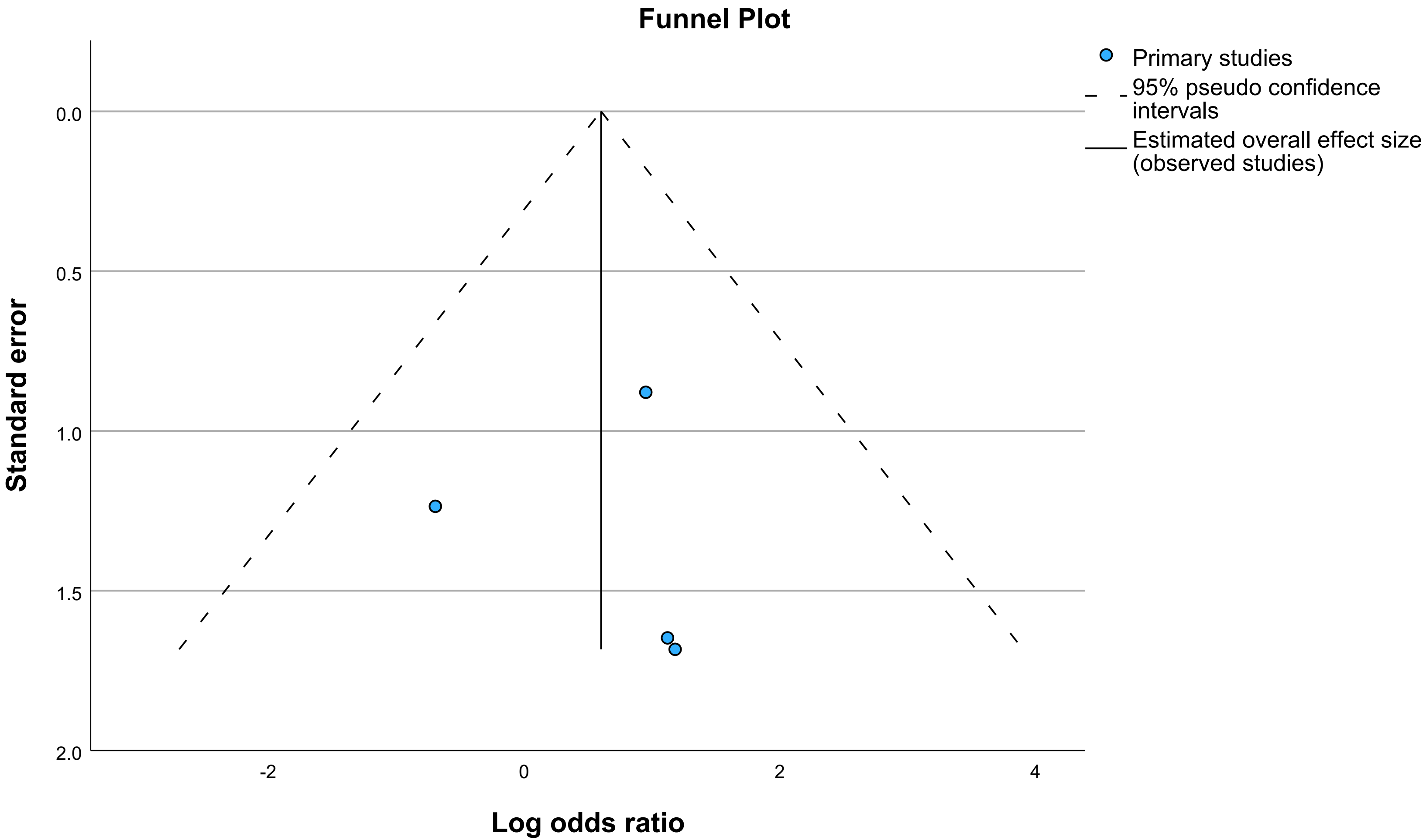
Funnel Plot
Primary studies
95% pseudo confidence intervals
Estimated overall effect size (observed studies)
Standard error
0.0
0.5
1.0
1.5
2.0
-2
0
2
4
Log odds ratio

Article title: Preoperative carbohydrate loading in elective colorectal surgery: postoperative complications and outcomes, a systematic review and meta-analysis

Journal: International Journal of Colorectal Disease

Authors: Aristotelis Nikitaras, Manousos-Georgios Pramateftakis, Konstantinos Perivoliotis, Sandra Maria Tsoti, Prokopis Christodoulou, Orestis Ioannidis, George Tzovaras

Corresponding author: Aristotelis Nikitaras, 1st Department of Surgery, Asklepieio General Hospital of Voula, Athens, Greece

Email: nikitaras.aristotelis@gmail.com

Online Resource 4: Funnel plots for all outcomes – First defecation funnel plot

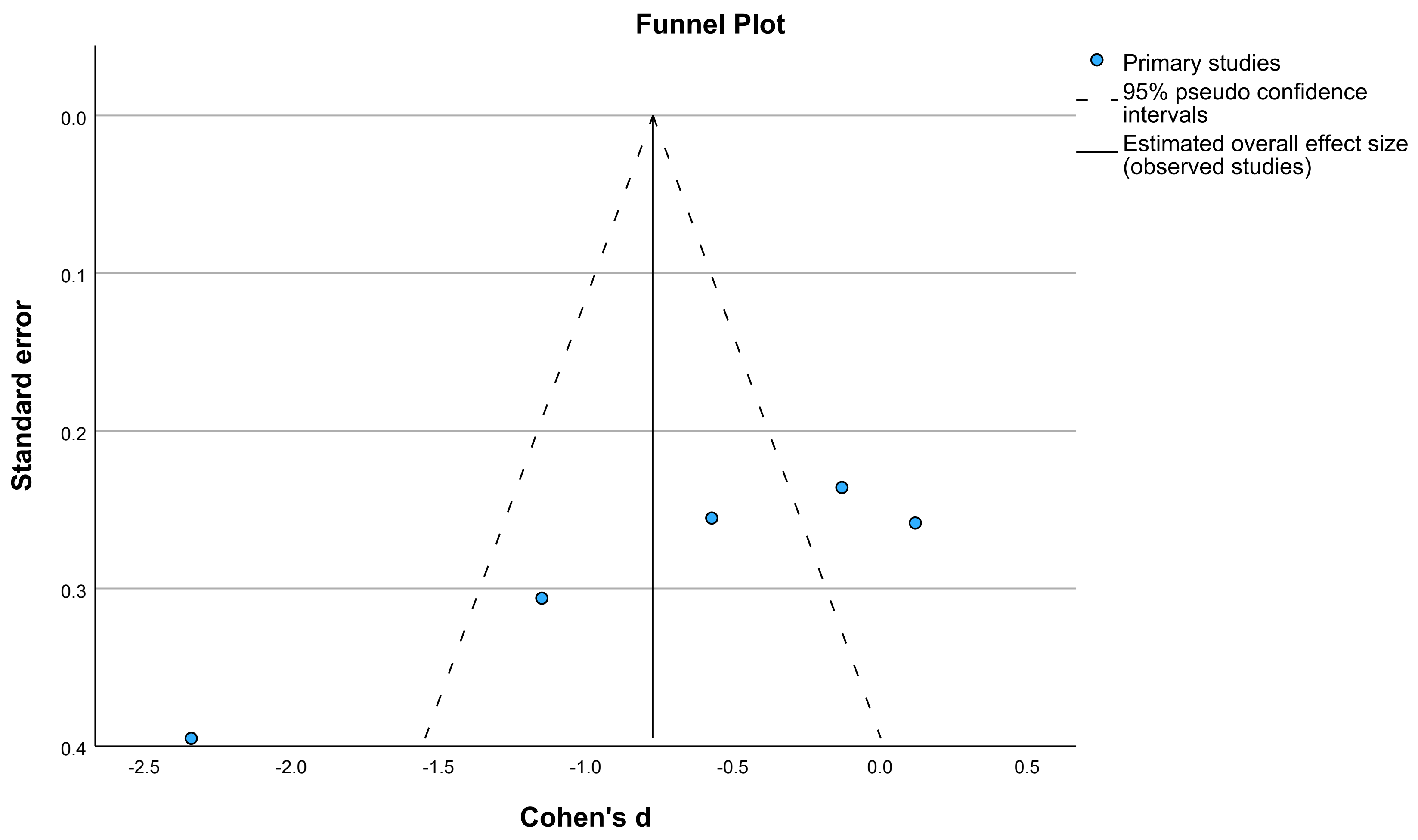

Funnel Plot
Primary studies
95% pseudo confidence intervals
Estimated overall effect size (observed studies)
Standard error
0.0
0.1
0.2
0.3
0.4
-2.5
-2.0
-1.5
-1.0
-0.5
0.0
0.5
Cohen's d

Article title: Preoperative carbohydrate loading in elective colorectal surgery: postoperative complications and outcomes, a systematic review and meta-analysis

Journal: International Journal of Colorectal Disease

Authors: Aristotelis Nikitaras, Manousos-Georgios Pramateftakis, Konstantinos Perivoliotis, Sandra Maria Tsoti, Prokopis Christodoulou, Orestis Ioannidis, George Tzovaras

Corresponding author: Aristotelis Nikitaras, 1st Department of Surgery, Asklepieio General Hospital of Voula, Athens, Greece

Email: nikitaras.aristotelis@gmail.com

Online Resource 4: Funnel plots for all outcomes – First flatus funnel plot

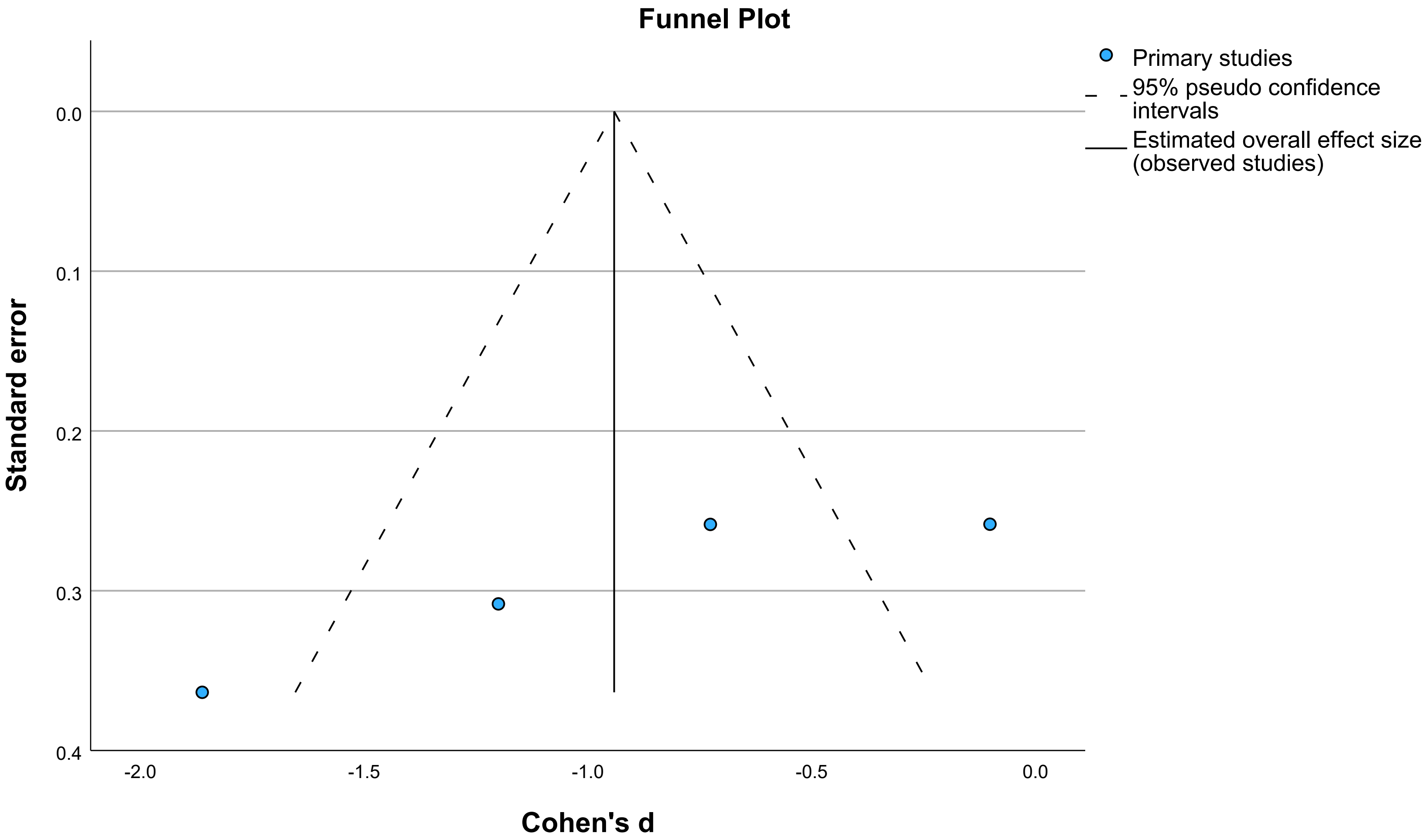

Funnel Plot
Primary studies
95% pseudo confidence intervals
Estimated overall effect size (observed studies)
Standard error
0.0
0.1
0.2
0.3
0.4
-2.0
-1.5
-1.0
-0.5
0.0
Cohen's d

Article title: Preoperative carbohydrate loading in elective colorectal surgery: postoperative complications and outcomes, a systematic review and meta-analysis

Journal: International Journal of Colorectal Disease

Authors: Aristotelis Nikitaras, Manousos-Georgios Pramateftakis, Konstantinos Perivoliotis, Sandra Maria Tsoti, Prokopis Christodoulou, Orestis Ioannidis, George Tzovaras

Corresponding author: Aristotelis Nikitaras, 1st Department of Surgery, Asklepieio General Hospital of Voula, Athens, Greece

Email: nikitaras.aristotelis@gmail.com

Online Resource 4: Funnel plots for all outcomes – Mobilisation funnel plot

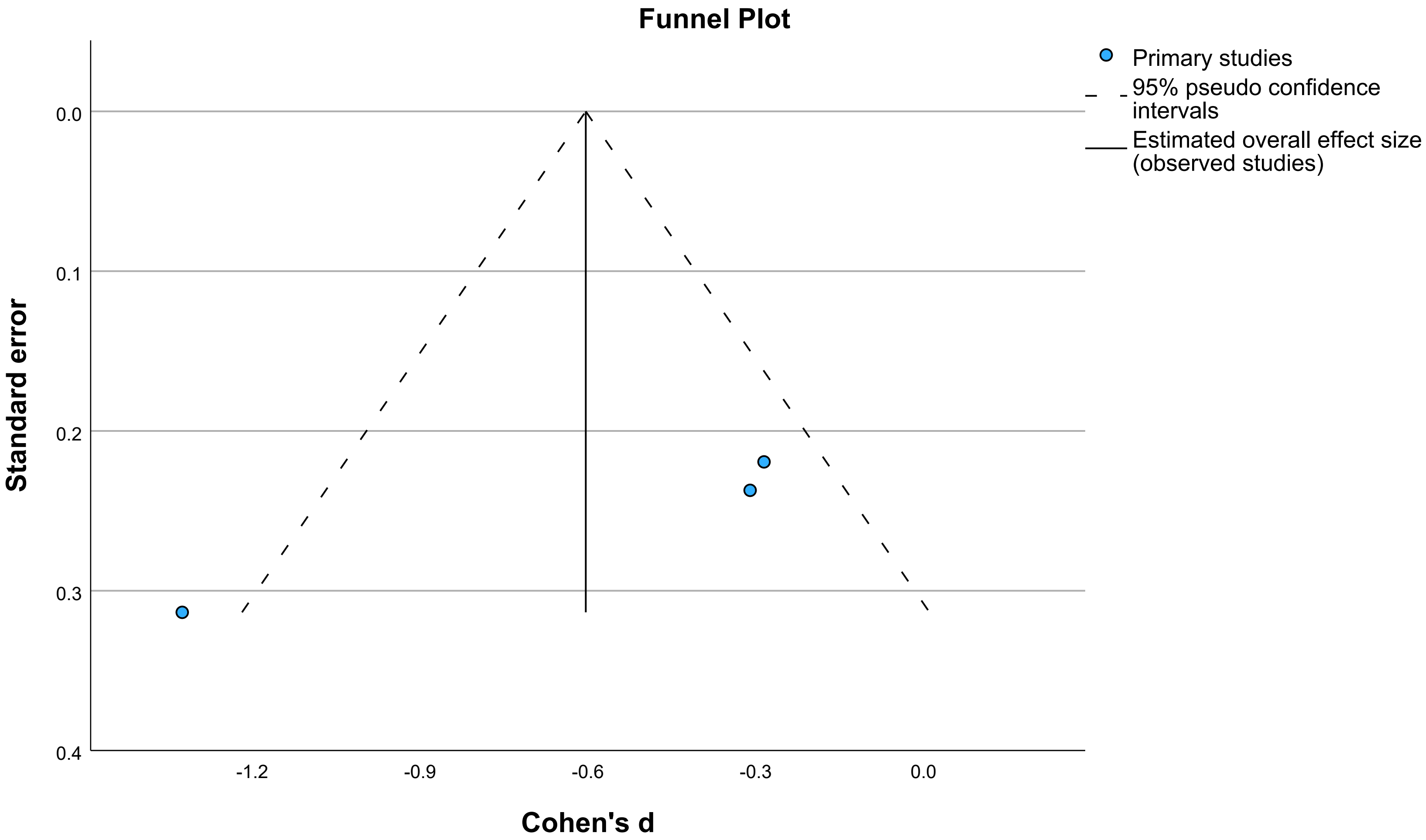
Funnel Plot
Primary studies
95% pseudo confidence intervals
Estimated overall effect size (observed studies)
Standard error
0.0
0.1
0.2
0.3
0.4
-1.2
-0.9
-0.6
-0.3
0.0
Cohen's d

Article title: Preoperative carbohydrate loading in elective colorectal surgery: postoperative complications and outcomes, a systematic review and meta-analysis

Journal: International Journal of Colorectal Disease

Authors: Aristotelis Nikitaras, Manousos-Georgios Pramateftakis, Konstantinos Perivoliotis, Sandra Maria Tsoti, Prokopis Christodoulou, Orestis Ioannidis, George Tzovaras

Corresponding author: Aristotelis Nikitaras, 1st Department of Surgery, Asklepieio General Hospital of Voula, Athens, Greece

Email: nikitaras.aristotelis@gmail.com

Online Resource 4: Funnel plots for all outcomes – Mortality funnel plot

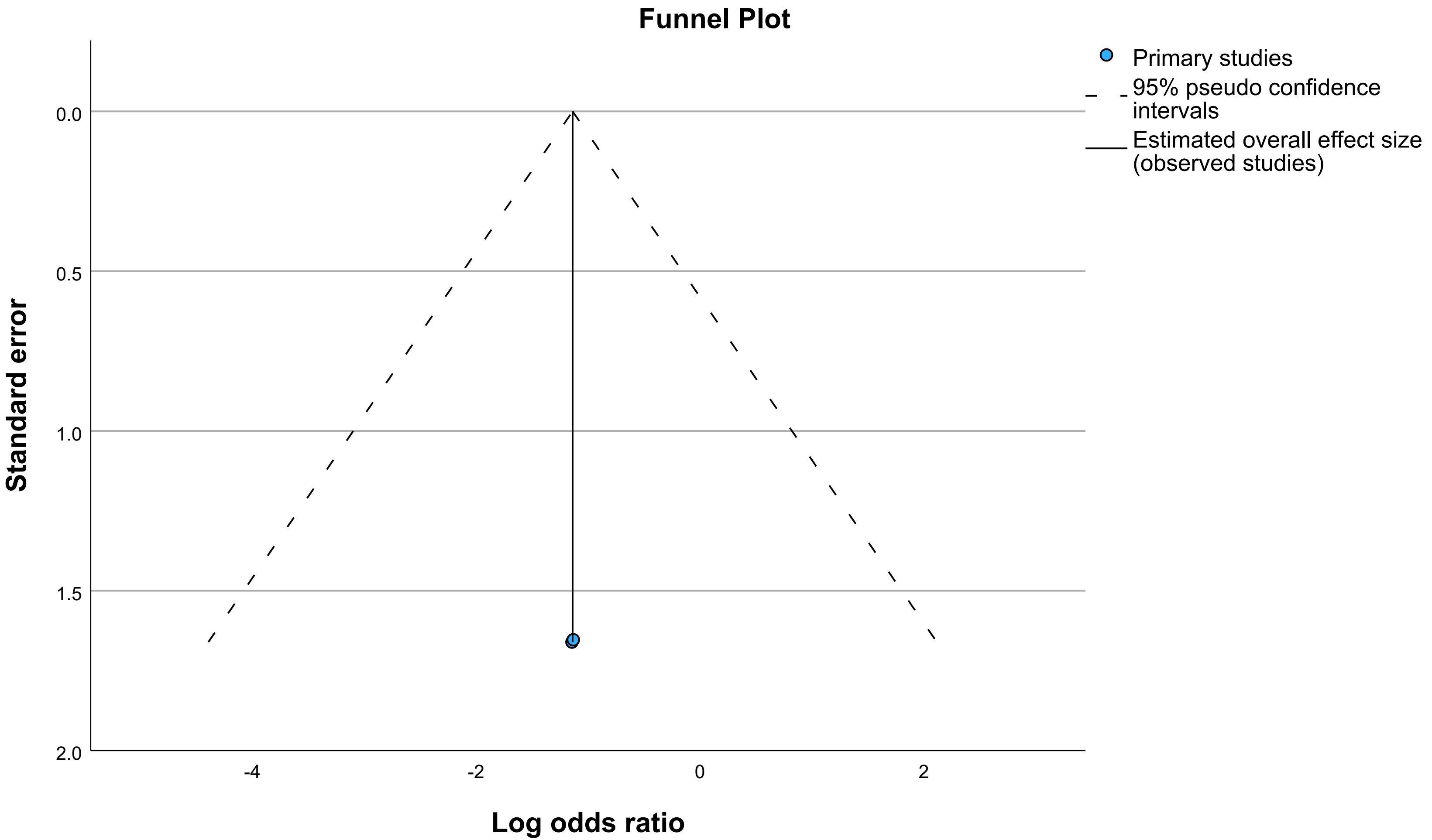
Funnel Plot
Primary studies
95% pseudo confidence intervals
Estimated overall effect size (observed studies)
Standard error
0.0
0.5
1.0
1.5
2.0
-4
-2
0
2
Log odds ratio
