# Supplementary material for: Preoperative carbohydrate loading in elective colorectal surgery: postoperative complications and outcomes, a systematic review and meta-analysis
Source: Int J Colorectal Dis. 2026 Apr 18;41(1):95. doi: 10.1007/s00384-026-05125-7 (PMC13222900; doi:10.1007/s00384-026-05125-7)
Supplement: Supplementary file 5 — Supplementary file5 Meta-regression bubble plots (age, BMI, male percentage, MIS percentage, publication year) (PDF 578 KB) [file 384_2026_5125_MOESM5_ESM.pdf]

Article title: Preoperative carbohydrate loading in elective colorectal surgery: postoperative complications and outcomes, a systematic review and meta-analysis

Journal: International Journal of Colorectal Disease

Authors: Aristotelis Nikitaras, Manousos-Georgios Pramateftakis, Konstantinos Perivoliotis, Sandra Maria Tsoti, Prokopis Christodoulou, Orestis Ioannidis, George Tzovaras

Corresponding author: Aristotelis Nikitaras, 1st Department of Surgery, Asklepieio General Hospital of Voula, Athens, Greece

Email: nikitaras.aristotelis@gmail.com

Online Resource 5: Meta-regression bubble plots – Age meta-regression

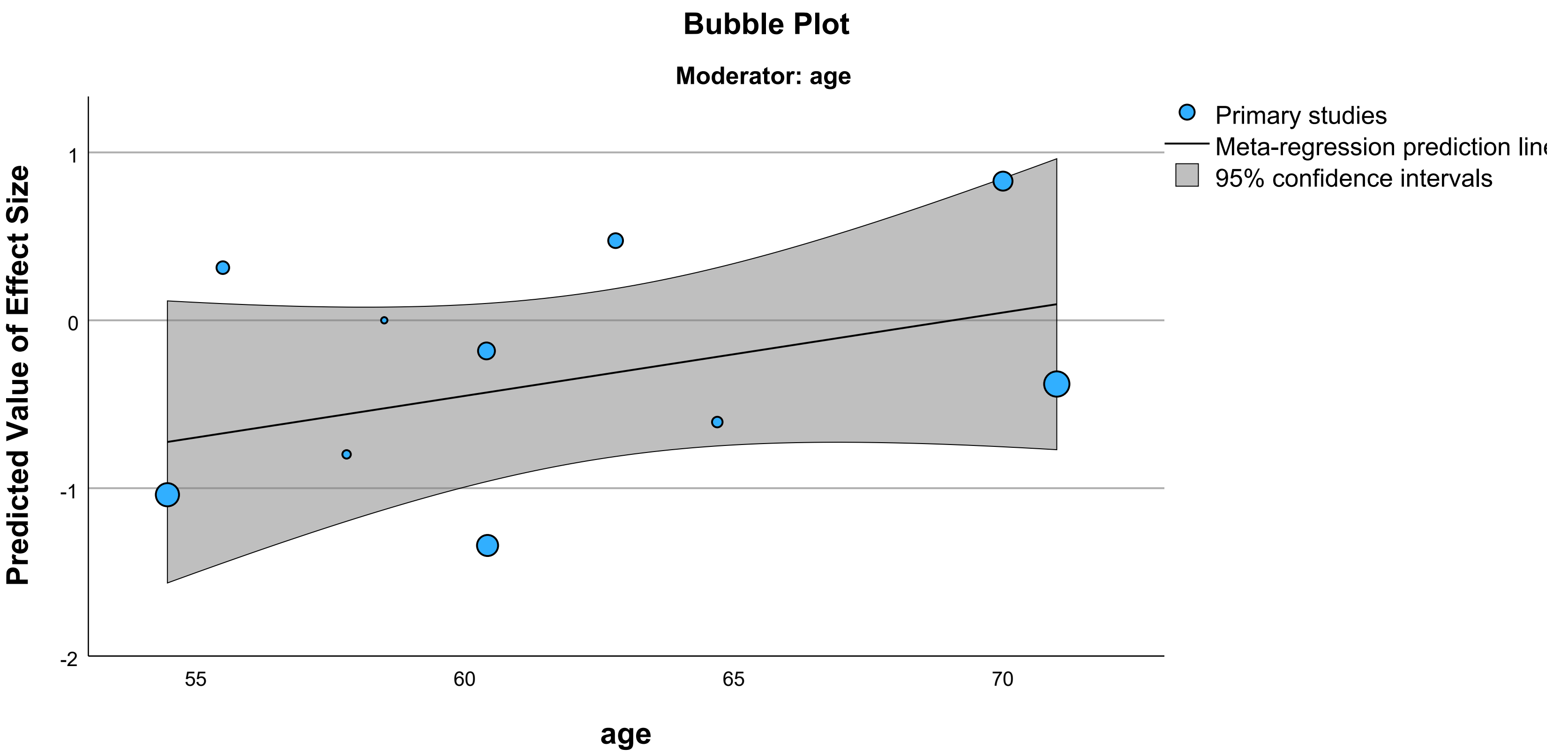

Model: Fixed-effects model

Weights: Inverse-variance

Confidence Intervals: Estimated based on t-distribution

Article title: Preoperative carbohydrate loading in elective colorectal surgery: postoperative complications and outcomes, a systematic review and meta-analysis

Journal: International Journal of Colorectal Disease

Authors: Aristotelis Nikitaras, Manousos-Georgios Pramateftakis, Konstantinos Perivoliotis, Sandra Maria Tsoti, Prokopis Christodoulou, Orestis Ioannidis, George Tzovaras

Corresponding author: Aristotelis Nikitaras, 1st Department of Surgery, Asklepieio General Hospital of Voula, Athens, Greece

Email: nikitaras.aristotelis@gmail.com

Online Resource 5: Meta-regression bubble plots – BMI meta-regression

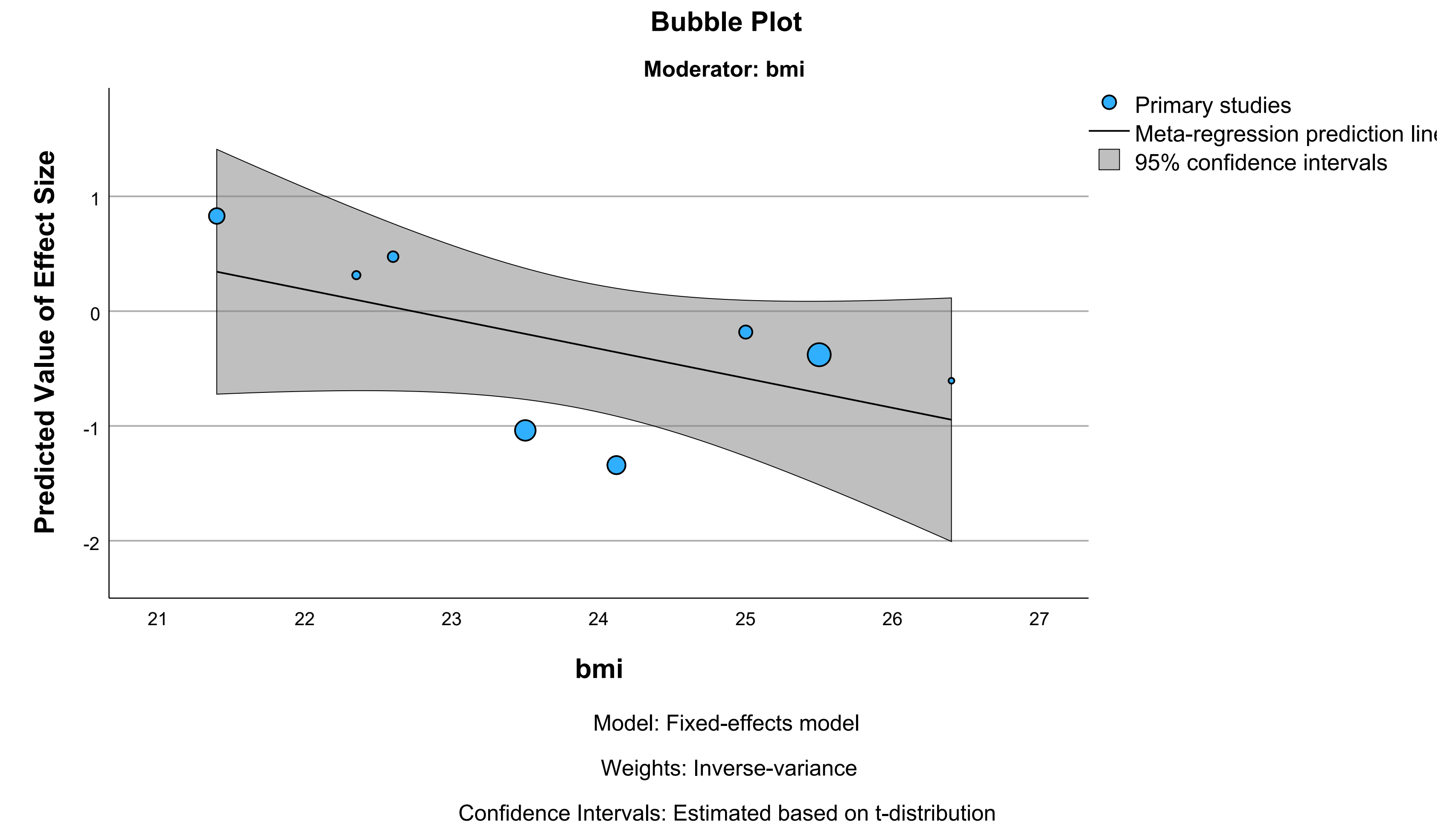

Bubble Plot
Moderator: bmi
Primary studies
Meta-regression prediction line
95% confidence intervals
Predicted Value of Effect Size
1
0
-1
-2
21
22
23
24
25
26
27
bmi
Model: Fixed-effects model
Weights: Inverse-variance
Confidence Intervals: Estimated based on t-distribution

Article title: Preoperative carbohydrate loading in elective colorectal surgery: postoperative complications and outcomes, a systematic review and meta-analysis

Journal: International Journal of Colorectal Disease

Authors: Aristotelis Nikitaras, Manousos-Georgios Pramateftakis, Konstantinos Perivoliotis, Sandra Maria Tsoti, Prokopis Christodoulou, Orestis Ioannidis, George Tzovaras

Corresponding author: Aristotelis Nikitaras, 1st Department of Surgery, Asklepieio General Hospital of Voula, Athens, Greece

Email: nikitaras.aristotelis@gmail.com

Online Resource 5: Meta-regression bubble plots – Male percentage meta-regression

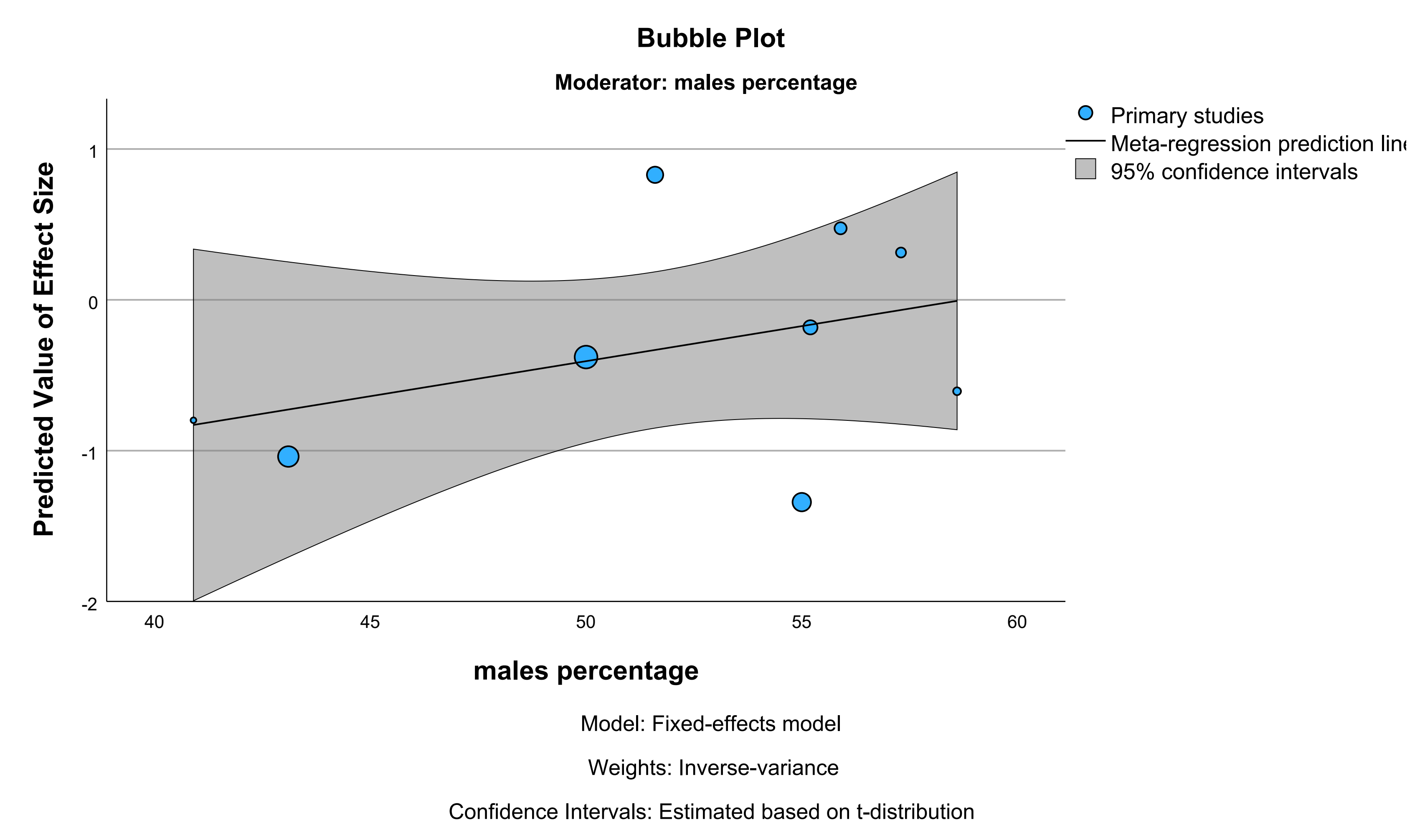
Bubble Plot
Moderator: males percentage
Primary studies
Meta-regression prediction line
95% confidence intervals
Predicted Value of Effect Size
1
0
-1
-2
40
45
50
55
60
males percentage
Model: Fixed-effects model
Weights: Inverse-variance
Confidence Intervals: Estimated based on t-distribution

Article title: Preoperative carbohydrate loading in elective colorectal surgery: postoperative complications and outcomes, a systematic review and meta-analysis

Journal: International Journal of Colorectal Disease

Authors: Aristotelis Nikitaras, Manousos-Georgios Pramateftakis, Konstantinos Perivoliotis, Sandra Maria Tsoti, Prokopis Christodoulou, Orestis Ioannidis, George Tzovaras

Corresponding author: Aristotelis Nikitaras, 1st Department of Surgery, Asklepieio General Hospital of Voula, Athens, Greece

Email: nikitaras.aristotelis@gmail.com

Online Resource 5: Meta-regression bubble plots – MIS percentage meta-regression

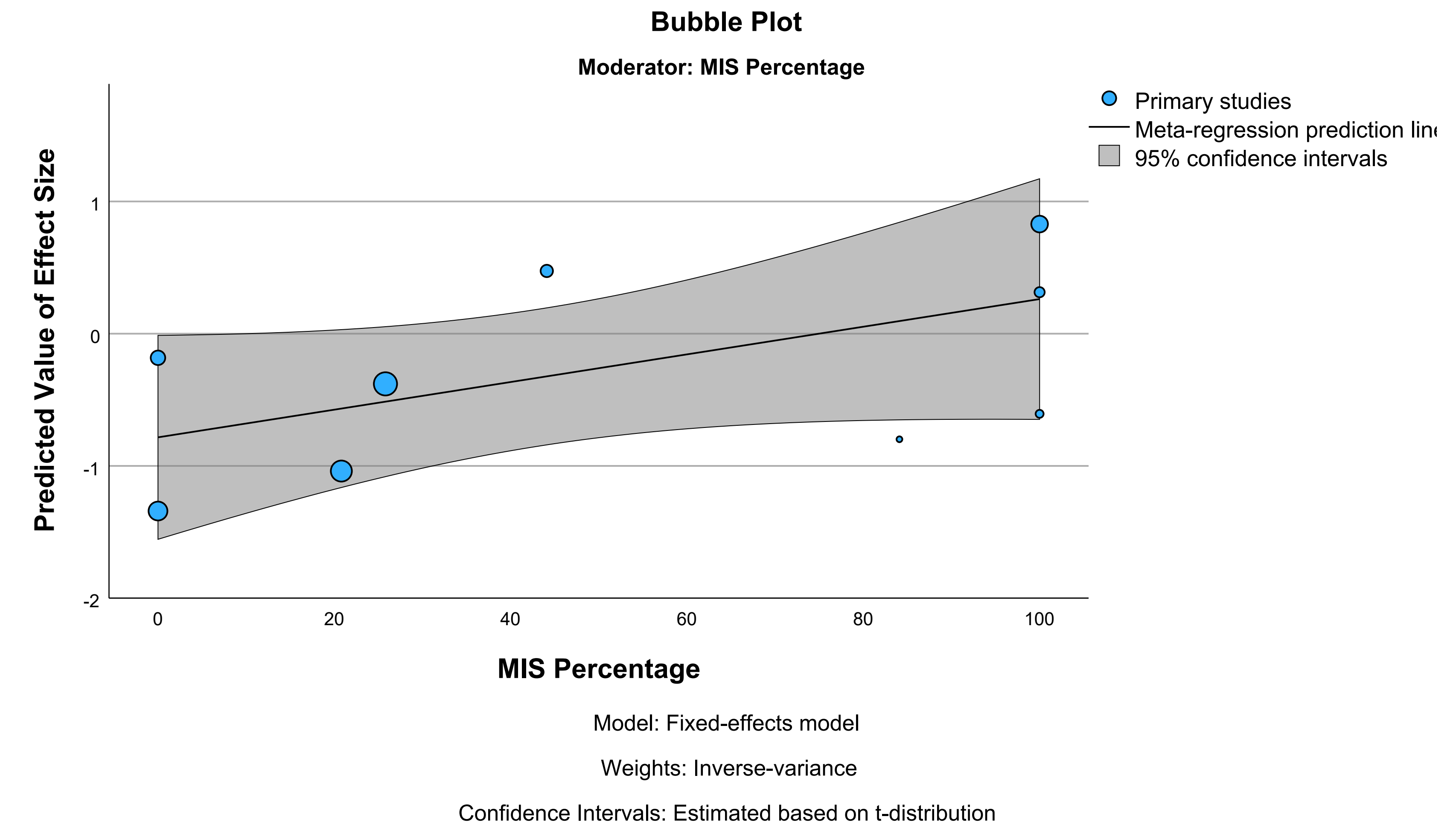
Bubble Plot
Moderator: MIS Percentage
Primary studies
Meta-regression prediction line
95% confidence intervals
Predicted Value of Effect Size
1
0
-1
-2
0
20
40
60
80
100
MIS Percentage
Model: Fixed-effects model
Weights: Inverse-variance
Confidence Intervals: Estimated based on t-distribution

Article title: Preoperative carbohydrate loading in elective colorectal surgery: postoperative complications and outcomes, a systematic review and meta-analysis

Journal: International Journal of Colorectal Disease

Authors: Aristotelis Nikitaras, Manousos-Georgios Pramateftakis, Konstantinos Perivoliotis, Sandra Maria Tsoti, Prokopis Christodoulou, Orestis Ioannidis, George Tzovaras

Corresponding author: Aristotelis Nikitaras, 1st Department of Surgery, Asklepieio General Hospital of Voula, Athens, Greece

Email: nikitaras.aristotelis@gmail.com

Online Resource 5: Meta-regression bubble plots – Publication year meta-regression

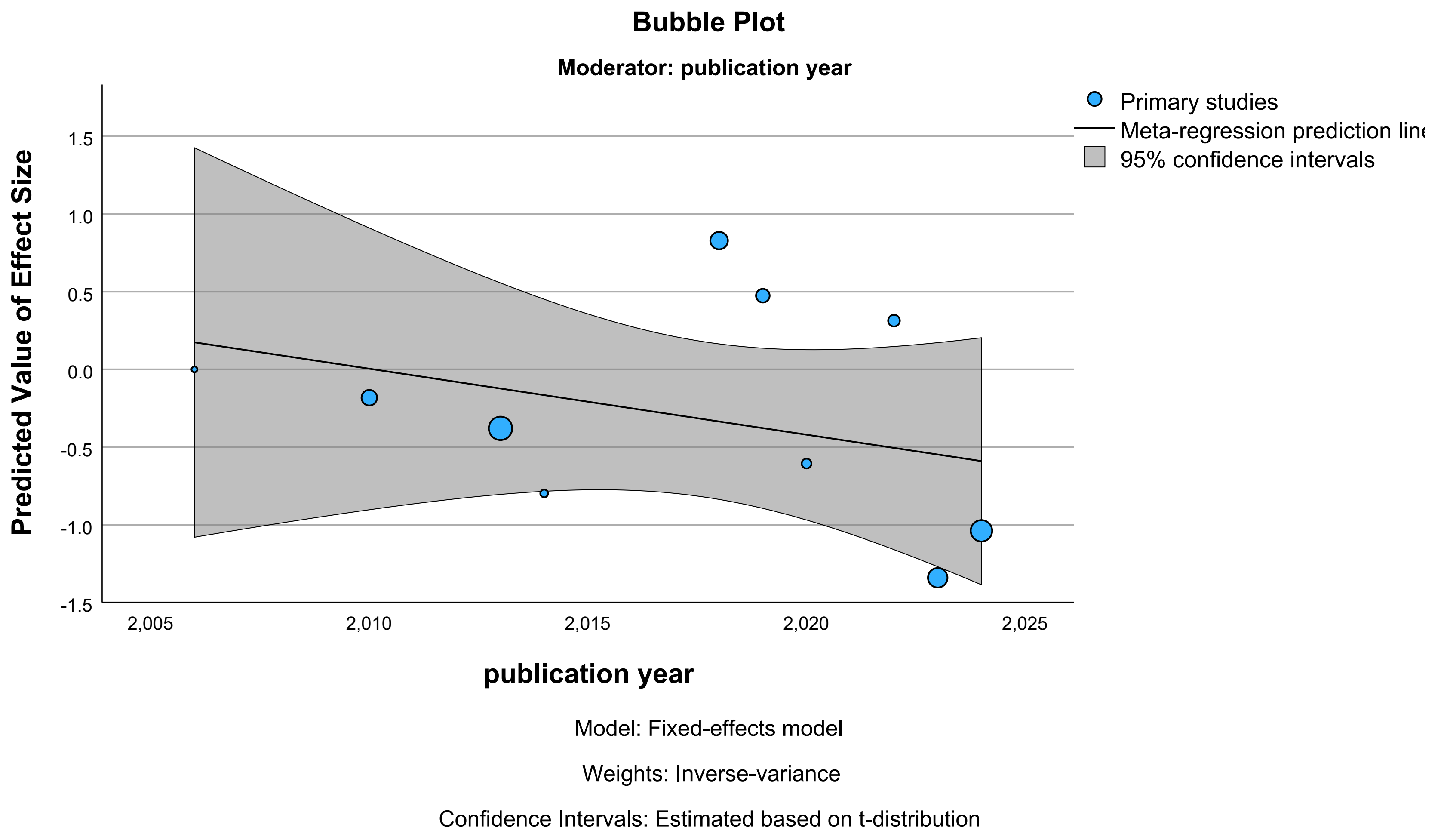
Bubble Plot
Moderator: publication year
Primary studies
Meta-regression prediction lin
95% confidence intervals
Predicted Value of Effect Size
1.5
1.0
0.5
0.0
-0.5
-1.0
-1.5
2,005
2,010
2,015
2,020
2,025
publication year
Model: Fixed-effects model
Weights: Inverse-variance
Confidence Intervals: Estimated based on t-distribution
