# Supplementary material for: Preoperative carbohydrate loading in elective colorectal surgery: postoperative complications and outcomes, a systematic review and meta-analysis
Source: Int J Colorectal Dis. 2026 Apr 18;41(1):95. doi: 10.1007/s00384-026-05125-7 (PMC13222900; doi:10.1007/s00384-026-05125-7)
Supplement: Supplementary file 6 — Supplementary file6 Subgroup plots (control type, number of centres) (PDF 220 KB) [file 384_2026_5125_MOESM6_ESM.pdf]

Article title: Preoperative carbohydrate loading in elective colorectal surgery: postoperative complications and outcomes, a systematic review and meta-analysis

Journal: International Journal of Colorectal Disease

Authors: Aristotelis Nikitaras, Manousos-Georgios Pramateftakis, Konstantinos Perivoliotis, Sandra Maria Tsoti, Prokopis Christodoulou, Orestis Ioannidis, George Tzovaras

Corresponding author: Aristotelis Nikitaras, 1st Department of Surgery, Asklepieio General Hospital of Voula, Athens, Greece

Email: nikitaras.aristotelis@gmail.com

Online Resource 6: Subgroup plots - Control type subgroup forest plot

Effect size of each study

Estimated overall effect size

Estimated overall confidence interval

Confidence interval of effect size

Overall effect size value

control	ID	OR	Lower	Upper	p-value	Weight	Weight (%)
Clear Fluids	J. Webster et al.	0.45	0.07	2.76	0.39	1.17	5.49
	Subgroup Overall	0.45	0.07	2.76	0.39		
Fasting	S. M. Kumar et al.	0.35	0.12	1.07	0.07	3.12	14.65
	Y. Deng et al.	1.37	0.29	6.53	0.69	1.57	7.39
	N. Rizvanovi et al. 2023	0.26	0.08	0.87	0.03	2.66	12.49
	S. E. Noblett et al.	1.00	0.06	18.08	1.00	0.46	2.15
	M. Kaška et al.	0.83	0.24	2.86	0.77	2.53	11.86
	Subgroup Overall	0.52	0.28	0.96	0.04		
Simple CHO	N. Karimian et al.	0.55	0.10	2.89	0.48	1.38	6.48
	Subgroup Overall	0.55	0.10	2.89	0.48		
Water	M. Wongyingsinn et al.	1.61	0.41	6.30	0.50	2.06	9.66
	P. Lidder et al.	0.68	0.25	1.87	0.46	3.80	17.86
	H. Hamamoto et al.	2.29	0.67	7.82	0.19	2.55	11.97
	Subgroup Overall	1.22	0.62	2.39	0.57		
Overall		0.72	0.47	1.11	0.13		

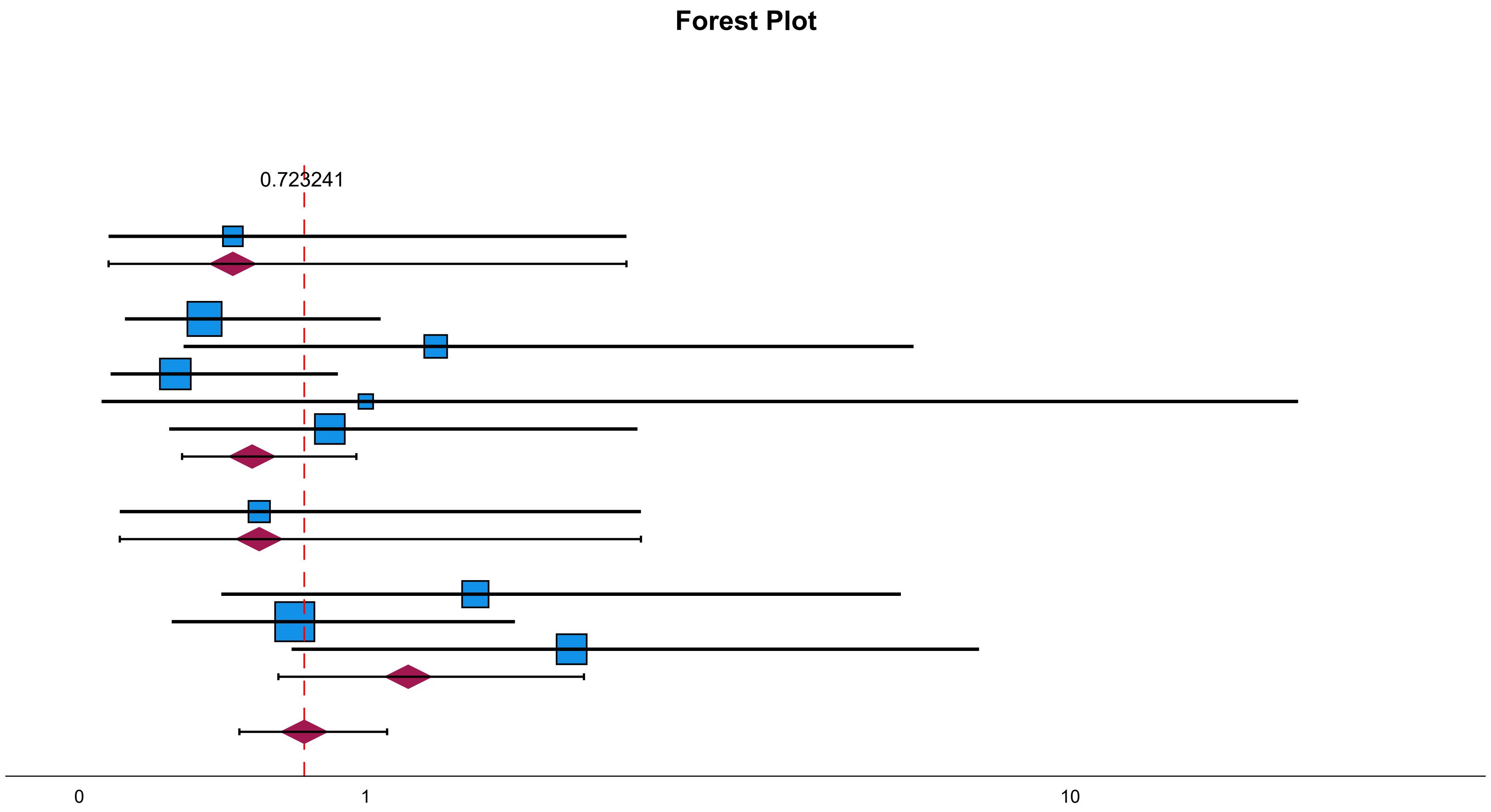

Model: Fixed-effects model

Heterogeneity: H-squared = 1.13, I-squared = 0.12

Homogeneity: Q = 10.17, df = 9, p-value = 0.34

Test of overall effect size: z = -1.50, p-value = 0.13

Test of between-subgroup homogeneity: Q = 3.78, df = 3, p-value = 0.29

Axis is shown using log scale

Article title: Preoperative carbohydrate loading in elective colorectal surgery: postoperative complications and outcomes, a systematic review and meta-analysis

Journal: International Journal of Colorectal Disease

Authors: Aristotelis Nikitaras, Manousos-Georgios Pramateftakis, Konstantinos Perivoliotis, Sandra Maria Tsoti, Prokopis Christodoulou, Orestis Ioannidis, George Tzovaras

Corresponding author: Aristotelis Nikitaras, 1st Department of Surgery, Asklepieio General Hospital of Voula, Athens, Greece

Email: nikitaras.aristotelis@gmail.com

Online Resource 6: Subgroup plots – Number of centres subgroup forest plot

Effect size of each study
Estimated overall effect size
Estimated overall confidence interval
Confidence interval of effect size
Overall effect size value

centers	ID	OR	Lower	Upper	p-value	Weight	Weight (%)
Multi	M. Kaška et al.	0.83	0.24	2.86	0.77	2.53	11.86
	Subgroup Overall	0.83	0.24	2.86	0.77		
Single	M. Wongyingsinn et al.	1.61	0.41	6.30	0.50	2.06	9.66
	P. Lidder et al.	0.68	0.25	1.87	0.46	3.80	17.86
	J. Webster et al.	0.45	0.07	2.76	0.39	1.17	5.49
	S. M. Kumar et al.	0.35	0.12	1.07	0.07	3.12	14.65
	Y. Deng et al.	1.37	0.29	6.53	0.69	1.57	7.39
	N. Rizvanovi et al. 2023	0.26	0.08	0.87	0.03	2.66	12.49
	S. E. Noblett et al.	1.00	0.06	18.08	1.00	0.46	2.15
	N. Karimian et al.	0.55	0.10	2.89	0.48	1.38	6.48
	H. Hamamoto et al.	2.29	0.67	7.82	0.19	2.55	11.97
	Subgroup Overall	0.71	0.45	1.12	0.14		
Overall		0.72	0.47	1.11	0.13		

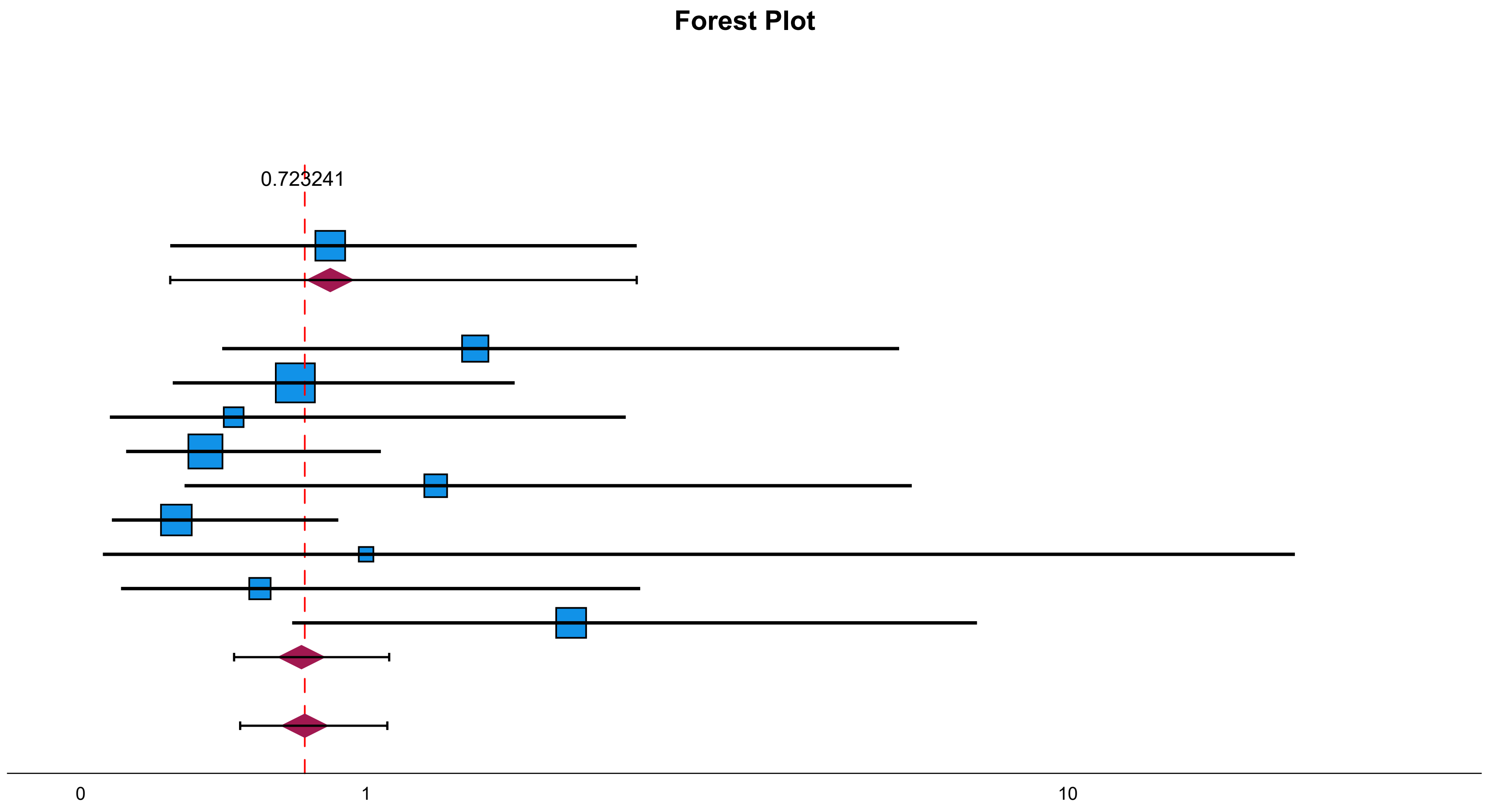

Model: Fixed-effects model

Heterogeneity: H-squared = 1.13, I-squared = 0.12

Homogeneity: Q = 10.17, df = 9, p-value = 0.34

Test of overall effect size: z = -1.50, p-value = 0.13

Test of between-subgroup homogeneity: Q = 0.06, df = 1, p-value = 0.81

Axis is shown using log scale
