# Supplementary material for: Preoperative carbohydrate loading in elective colorectal surgery: postoperative complications and outcomes, a systematic review and meta-analysis
Source: Int J Colorectal Dis. 2026 Apr 18;41(1):95. doi: 10.1007/s00384-026-05125-7 (PMC13222900; doi:10.1007/s00384-026-05125-7)
Supplement: Supplementary file 7 — Supplementary file7 PRISMA 2020 checklist (PDF 231 KB) [file 384_2026_5125_MOESM7_ESM.pdf]

PRISMA 2020 Checklist

Article title: Preoperative carbohydrate loading in elective colorectal surgery: postoperative complications and outcomes, a systematic review and meta-analysis

Journal: International Journal of Colorectal Disease

Authors: Aristotelis Nikitaras, Manousos-Georgios Pramateftakis, Konstantinos Perivoliotis, Sandra Maria Tsoti, Prokopis Christodoulou, Orestis Ioannidis, George Tzovaras

Corresponding author: Aristotelis Nikitaras, 1st Department of Surgery, Asklepieio General Hospital of Voula, Athens, Greece

Email: nikitaras.aristotelis@gmail.com

Online Resource 7: PRISMA 2020 checklist

PRISMA

PRISMA 2020 Checklist

Section and Topic	Item #	Checklist item	Location where item is reported
**TITLE**			
Title	1	Identify the report as a systematic review.	Page 1
**ABSTRACT**			
Abstract	2	See the PRISMA 2020 for Abstracts checklist.	Page 2
**INTRODUCTION**			
Rationale	3	Describe the rationale for the review in the context of existing knowledge.	Page 3, paragraphs 1-2
Objectives	4	Provide an explicit statement of the objective(s) or question(s) the review addresses.	Introduction, Page 4
**METHODS**			
Eligibility criteria	5	Specify the inclusion and exclusion criteria for the review and how studies were grouped for the syntheses.	Eligibility Criteria, Page 4
Information sources	6	Specify all databases, registers, websites, organisations, reference lists and other sources searched or consulted to identify studies. Specify the date when each source was last searched or consulted.	Literature search, Page 5
Search strategy	7	Present the full search strategies for all databases, registers and websites, including any filters and limits used.	Literature search, Page 5 and Online Resource 1
Selection process	8	Specify the methods used to decide whether a study met the inclusion criteria of the review, including how many reviewers screened each record and each report retrieved, whether they worked independently, and if applicable, details of automation tools used in the process.	Study and data collection, Page 5
Data collection process	9	Specify the methods used to collect data from reports, including how many reviewers collected data from each report, whether they worked independently, any processes for obtaining or confirming data from study investigators, and if applicable, details of automation tools used in the process.	Study and data collection, Page 5
Data items	10a	List and define all outcomes for which data were sought. Specify whether all results that were compatible with each outcome domain in each study were sought (e.g. for all measures, time points, analyses), and if not, the methods used to decide which results to collect.	Outcomes, Page 4
	10b	List and define all other variables for which data were sought (e.g. participant and intervention characteristics, funding sources). Describe any assumptions made about any missing or unclear information.	Study characteristics, Pages 7–8 (Table 1 and Online Resource 2)
Study risk of bias assessment	11	Specify the methods used to assess risk of bias in the included studies, including details of the tool(s) used, how many reviewers assessed each study and whether they worked independently, and if applicable, details of automation tools used in the process.	Study and data collection, Page 5
Effect measures	12	Specify for each outcome the effect measure(s) (e.g. risk ratio, mean difference) used in the synthesis or presentation of results.	Statistical analysis, Page 5
Synthesis methods	13a	Describe the processes used to decide which studies were eligible for each synthesis (e.g. tabulating the study intervention characteristics and comparing against the planned groups for each synthesis (item #5)).	Methods, Pages 4–5 and Results, Table 1 (Page 7)
	13b	Describe any methods required to prepare the data for presentation or synthesis, such as handling of missing summary statistics, or data conversions.	Statistical analysis, Page 5
	13c	Describe any methods used to tabulate or visually display results of individual studies and syntheses.	Table 1, Table 2,

Section and Topic	Item #	Checklist item	Location where item is reported
			Figure 2, Supplementary Material
	13d	Describe any methods used to synthesize results and provide a rationale for the choice(s). If meta-analysis was performed, describe the model(s), method(s) to identify the presence and extent of statistical heterogeneity, and software package(s) used.	Statistical analysis, Page 5
	13e	Describe any methods used to explore possible causes of heterogeneity among study results (e.g. subgroup analysis, meta-regression).	Statistical analysis, Page 5
	13f	Describe any sensitivity analyses conducted to assess robustness of the synthesized results.	Results – Overall postoperative complications, Page 9 and Online Resource 8
Reporting bias assessment	14	Describe any methods used to assess risk of bias due to missing results in a synthesis (arising from reporting biases).	Risk of bias across studies, Page 9
Certainty assessment	15	Describe any methods used to assess certainty (or confidence) in the body of evidence for an outcome.	No formal certainty framework (e.g., GRADE) was applied; certainty considerations are discussed narratively in the Limitations section (Page 13).
**RESULTS**			
Study selection	16a	Describe the results of the search and selection process, from the number of records identified in the search to the number of studies included in the review, ideally using a flow diagram.	Study selection, Page 6
	16b	Cite studies that might appear to meet the inclusion criteria, but which were excluded, and explain why they were excluded.	Study selection, Page 6
Study characteristics	17	Cite each included study and present its characteristics.	Study characteristics, Pages 7–8 (Table 1)
Risk of bias in studies	18	Present assessments of risk of bias for each included study.	Risk of bias within studies, Page 8 and Supplementary Material
Results of individual studies	19	For all outcomes, present, for each study: (a) summary statistics for each group (where appropriate) and (b) an effect estimate and its precision (e.g. confidence/credible interval), ideally using structured tables or plots.	Outcomes, Pages 8–9 and Supplementary Material
Results of syntheses	20a	For each synthesis, briefly summarise the characteristics and risk of bias among contributing studies.	Pages 7–10 and Supplementary Material
	20b	Present results of all statistical syntheses conducted. If meta-analysis was done, present for each the summary estimate and its precision	Outcomes, Pages 8–

PRISMA 2020 Checklist

Section and Topic	Item #	Checklist item	Location where item is reported
		(e.g. confidence/credible interval) and measures of statistical heterogeneity. If comparing groups, describe the direction of the effect.	9 and Supplementary Material
	20c	Present results of all investigations of possible causes of heterogeneity among study results.	Outcomes, Pages 8–9 and Supplementary Material
	20d	Present results of all sensitivity analyses conducted to assess the robustness of the synthesized results.	Results – Overall postoperative complications, Page 9 and Online Resource 8
Reporting biases	21	Present assessments of risk of bias due to missing results (arising from reporting biases) for each synthesis assessed.	Risk of bias across studies, Page 9
Certainty of evidence	22	Present assessments of certainty (or confidence) in the body of evidence for each outcome assessed.	Narrative certainty considerations are presented in the Limitations section (Page 13)
**DISCUSSION**			
Discussion	23a	Provide a general interpretation of the results in the context of other evidence.	Discussion, Pages 11–12
	23b	Discuss any limitations of the evidence included in the review.	Limitations, Page 13
	23c	Discuss any limitations of the review processes used.	Limitations, Page 13
	23d	Discuss implications of the results for practice, policy, and future research.	Discussion and Conclusion, Pages 11–14
**OTHER INFORMATION**			
Registration and protocol	24a	Provide registration information for the review, including register name and registration number, or state that the review was not registered.	Study Protocol, Page 4
	24b	Indicate where the review protocol can be accessed, or state that a protocol was not prepared.	Study Protocol, Page 4
	24c	Describe and explain any amendments to information provided at registration or in the protocol.	Not applicable, as the review was not registered and no prior protocol was prepared.
Support	25	Describe sources of financial or non-financial support for the review, and the role of the funders or sponsors in the review.	Statements and Declarations – Funding, Page 14
Competing	26	Declare any competing interests of review authors.	Statements and

Section and Topic	Item #	Checklist item	Location where item is reported
interests			Declarations – Competing interests, Page 14
Availability of data, code and other materials	27	Report which of the following are publicly available and where they can be found: template data collection forms; data extracted from included studies; data used for all analyses; analytic code; any other materials used in the review.	Statements and Declarations – Data availability, Page 14

From: Page MJ, McKenzie JE, Bossuyt PM, Boutron I, Hoffmann TC, Mulrow CD, et al. The PRISMA 2020 statement: an updated guideline for reporting systematic reviews. BMJ 2021;372:n71. doi: 10.1136/bmj.n71. This work is licensed under CC BY 4.0. To view a copy of this license, visit https://creativecommons.org/licenses/by/4.0/
