# Supplementary material for: Preoperative carbohydrate loading in elective colorectal surgery: postoperative complications and outcomes, a systematic review and meta-analysis
Source: Int J Colorectal Dis. 2026 Apr 18;41(1):95. doi: 10.1007/s00384-026-05125-7 (PMC13222900; doi:10.1007/s00384-026-05125-7)
Supplement: Supplementary file 8 — Supplementary file8 Sensitivity analyses (leave-one-out) and follow-up duration subgroup forest plot (30-day follow-up vs non–30-day follow-up) (PDF 157 KB) [file 384_2026_5125_MOESM8_ESM.pdf]

Article title: Preoperative carbohydrate loading in elective colorectal surgery: postoperative complications and outcomes, a systematic review and meta-analysis

Journal: International Journal of Colorectal Disease

Authors: Aristotelis Nikitaras, Manousos-Georgios Pramateftakis, Konstantinos Perivoliotis, Sandra Maria Tsoti, Prokopis Christodoulou, Orestis Ioannidis, George Tzovaras

Corresponding author: Aristotelis Nikitaras, 1st Department of Surgery, Asklepieio General Hospital of Voula, Athens, Greece

Email: nikitaras.aristotelis@gmail.com

Online Resource 8: Sensitivity analyses (leave-one-out) and follow-up duration subgroup forest plot (30-day follow-up vs non–30-day follow-up)

Sensitivity analyses (leave-one-out)

Study	Effect Size	95%CI	P	I^2	Q test p
H. Hamamoto et al.	0.62	0.39,0.97	0.04	0%	0.62
J. Webster et al.	0.74	0.48,1.15	0.18	19%	0.28
M. Kaška et al.	0.71	0.45,1.11	0.13	20%	0.26
M. Wongyingsinn et al.	0.66	0.42,1.04	0.07	8%	0.37
N. Karimian et al.	0.73	0.47,1.14	0.17	20%	0.27
N. Rizvanović et al. 2023	0.83	0.53,1.31	0.43	0%	0.54
P. Lidder et al.	0.73	0.46,1.17	0.19	21%	0.26
S. E. Noblett et al.	0.72	0.47,1.10	0.13	21%	0.26
S. M. Kumar et al.	0.81	0.51,1.29	0.38	3%	0.41
Y. Deng et al.	0.69	0.44,1.07	0.09	15%	0.31

Forest Plot

Effect size of each study | Confidence interval of effect size
Estimated overall effect size | Overall effect size value
No-effect value | Estimated overall confidence interva

followup	ID	OR	Lower	Upper	p-value	Weight	Weight (%)
Not 30 days follow up	J. Webster et al.	0.45	0.07	2.76	0.39	1.17	5.49
	S. M. Kumar et al.	0.35	0.12	1.07	0.07	3.12	14.65
	Y. Deng et al.	1.37	0.29	6.53	0.69	1.57	7.39
	S. E. Noblett et al.	1.00	0.06	18.08	1.00	0.46	2.15
	M. Kaška et al.	0.83	0.24	2.86	0.77	2.53	11.86
	H. Hamamoto et al.	2.29	0.67	7.82	0.19	2.55	11.97
	Subgroup Overall	0.84	0.47	1.50	0.55		
30 days follow up	M. Wongyingsinn et al.	1.61	0.41	6.30	0.50	2.06	9.66
	P. Lidder et al.	0.68	0.25	1.87	0.46	3.80	17.86
	N. Rizvanovi et al. 2023	0.26	0.08	0.87	0.03	2.66	12.49
	N. Karimian et al.	0.55	0.10	2.89	0.48	1.38	6.48
	Subgroup Overall	0.61	0.33	1.14	0.12		
Overall		0.72	0.47	1.11	0.13		

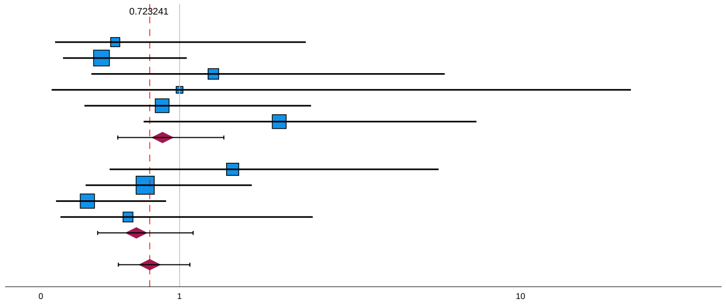

Model: Fixed-effects model
Heterogeneity: H-squared = 1.13, I-squared = 0.12
Homogeneity: Q = 10.17, df = 9, p-value = 0.34
Test of overall effect size: z = -1.50, p-value = 0.13
Test of between-subgroup homogeneity: Q = 0.52, df = 1, p-value = 0.47
Axis is shown using log scale
